# Current State of Computational Modeling of Nanohelicenes

**DOI:** 10.3390/nano13162295

**Published:** 2023-08-09

**Authors:** Vitaly V. Porsev, Robert A. Evarestov

**Affiliations:** Quantum Chemistry Department, Saint-Petersburg State University, St Petersburg 199034, Russia

**Keywords:** helicene, nanohelicene, graphene spiral, graphene helicoid, helical graphene nanoribbon, helical graphene, DFT, DFTB, molecular dynamics, helical periodicity, line symmetry groups

## Abstract

This review considers the works that focus on various aspects of the theoretical description of nanohelicenes (other equivalent names are graphene spirals, graphene helicoid, helical graphene nanoribbon, or helical graphene)—a promising class of one-dimensional nanostructures. The intrinsic helical topology and continuous π-system lead to the manifestation of unique optical, electronic, and magnetic properties that are also highly dependent on axial and torsion strains. In this paper, it was shown that the properties of nanohelicenes are mainly associated with the peripheral modification of the nanohelicene ribbon. We have proposed a nomenclature that enables the classification of all nanohelicenes as modifications of some prototype classes.

## 1. Introduction

Helical structures formed at the molecular level have attracted the attention of researchers for a long time. Many molecular structures in living organisms exist in the form of helices, in particular DNA and RNA molecules and various peptides. Such molecules are natural polymers. The successive development of methods of polymer organic chemistry has led to the possibility of the synthesis of artificial stereoregular polymers, the threads of which are also ordered in a spiral manner (helical polymers) [1,2,3]. The spiral topology opens up great opportunities for various technological applications regarding helical polymers. In particular, the circularly polarized luminescence (CPL) of cis-cisoid polyene-based polymers [4], the magneto-optical properties of helical poly-3-(alkylsulfone)thiophene [5], various stimuli-responsive devices as sensors [6], asymmetric catalysis and chiral recognition [2], and chirality-induced spin selectivity (CISS) [7] should be mentioned.

The development of experimental methods for the synthesis of nanostructures that are much more extended in one dimension than in the other two (for brevity, we call such objects one-dimensional, 1D) made it possible to obtain nano objects of various morphologies and structures, including helical ones [8]. Among the most frequently used synthetic methods that allow for 1D helical objects to be obtained are the following: (i) the screw-dislocation-driven growth of 1D crystals [8] and (ii) various methods based on the addition of a chiral component to the reaction mixture (template-assisted synthesis) [9]. The 1D nanostructures with intrinsic chirality can be noted separately. The existence of such 1D crystals is due to the fact that the symmetry of the initial 3D crystals of these compounds is described by the Sohncke symmetry groups [10]. Among such structures, recently synthesized ultrathin tellurium nanorods can be mentioned [11].

**Figure 1:**
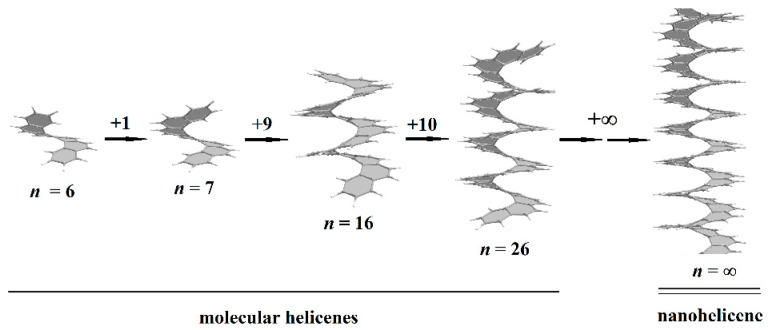
Molecular [*n*]helicenes and nanohelicene. From left to right: [6]helicene, [7]helicene, [16]helicene, [26]helicene, and [∞]helicene = nanohelicene. Gray and white spheres are carbon and hydrogen atoms, respectively.

Helical 1D nanostructures, here referred to as *nanohelicenes* [12], with both helical polymers and nanorods with intrinsic chirality can be considered. In molecular [*n*]helicenes, the number *n* denotes the number of ortho-fused benzene rings [13]. If *n* tends to infinity, then a polymeric [∞]helicene will be obtained, which can be considered as a ribbon infinitely turned along the helical axis (Figure 1). By considering [∞]helicene as the basic nanohelicene, it is possible to construct many modifications that will have different properties. At the same time, all nanohelicenes will have two main properties—a continuous π-system, and a helical topology.

Further, the consideration of nanohelicenes as “graphite” nanorods with a screw dislocation [14,15] makes it possible to understand these objects as structures formed by a graphene ribbon of a special morphology, which allows it to helically turn around a screw axis.

**Figure 2:**
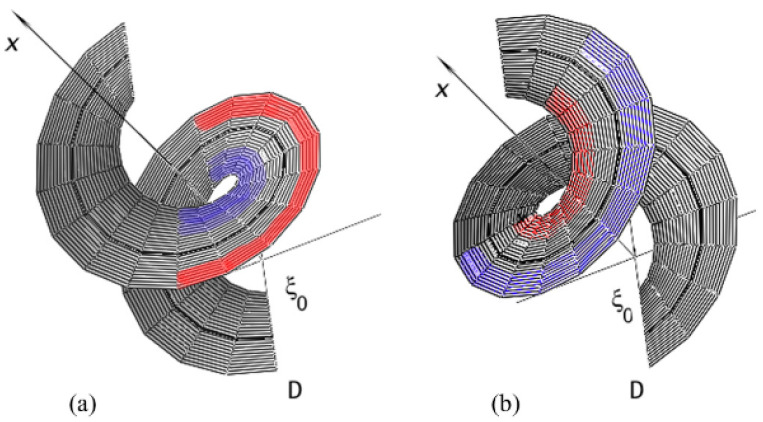
Examples of a conventional graphene nanoribbon forming helicoids with (**a**) left-handed and (**b**) right-handed spirals. Reprinted with permission from Ref. [20]. (Copyright 2015 American Physical Society).

Therefore, other names are also used for nanohelicenes: “graphene spiral” [16], “graphene helicoid” [17], “helical graphene nanoribbon” [18], and “helical graphene” [19]. In addition, conventional graphene nanoribbons can be twisted into “helicoidal graphene nanoribbons” [20], which are qualitatively different from the objects considered here (Figure 2).

Thus, the results obtained to date for various classes of nanohelicenes will be the focus of this review. In addition to the results of theoretical studies, the experimental data necessary for understanding the current level of synthetic capabilities and the prospects for using the systems under consideration will be reviewed.

First, the results of the theoretical studies obtained for molecular helicenes as oligomers of nanohelicenes will be considered. Then the original nomenclature of the prototype classes of nanohelicenes will be presented. Next, the current state of experimental results in the synthesis of the nanohelicenes will be described. Further, the theoretical results obtained by the methods of molecular dynamics (MDs) and density functional theory in semi-empirical tight-binding approximation (DFTB) and ab initio (DFT) variants will be reviewed. Next, we will consider the results of the calculations by using the DFT method, taking into account the helical periodicity of nanohelicenes. Finally, the main conclusions are presented in Section 7.

## 2. Theoretical Consideration of Molecular Helicenes and Their Properties

Molecular helicenes have a long story. In 1956, the [6]helicene was synthesized [21], which can be considered as one coil of the [∞]helicene. The synthesis of helicenes with two coils began in 1967 with the synthesis of the [7]helicene [22]. In 2015, the [14]helicene was obtained [23], i.e., helicene with three incomplete coils, and, to date, helicene with maximum *n* (Figure 1) remains to be obtained experimentally. It can be assumed that a further increase in *n* is a difficult synthetic problem, which is due to significant steric hindrance existing for inner atoms. However, there are some experimental data [24] that enable one to speculate on the presence of helicenes in interstellar space up to the [26]helicene (with more than four coils), which can be explained on the basis of the principles of minimum energy and polarizability (Figure 1) [25,26,27].

**Figure 3:**
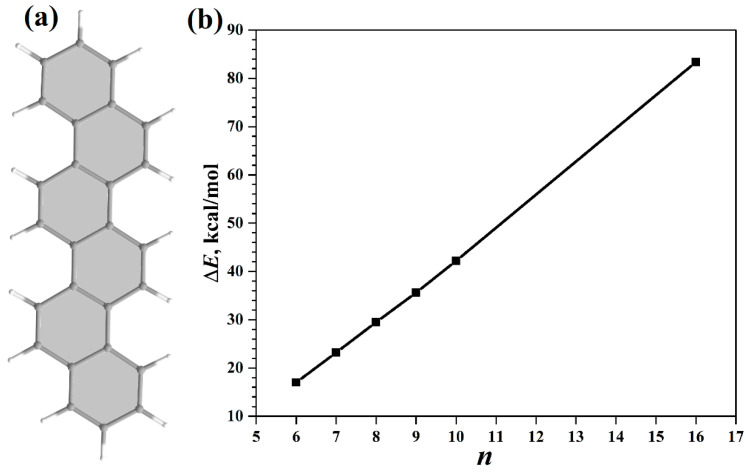
(**a**) [6]phenacene (the corresponding [6]helicene, see Figure 1). Gray and white spheres are carbon and hydrogen atoms, respectively. (**b**) The ∆*E*(*n*) dependence according to data from Ref. [31].

Thus, at the moment, the properties of helicenes with a further increase in *n*, and the approximation of properties to *n* → ∞ can only be estimated based on quantum chemical calculations. The first quantum chemical calculations (at the level of semi-empirical CNDO/S, PM3, and the HF method) of the helicenes appeared quite a long time ago and dealt with the structure of helicenes, racemization barriers, and chiroptical properties [28,29,30]. In [31], the effect of the distortion of the π-system due to twisting was theoretically investigated by comparing the energy characteristics of [*n*]helicenes and their planar counterparts, [*n*]phenacenes (Figure 3a). The calculations were carried out at the level of HF/6-31G* and B3LYP/6-31G* at *n* = 6–10 and *n* = 16 and in the more powerful 6-311G** basis set for *n* = 6–10.

The dependence of the difference, Δ*E*, between the energies of helicenes and phenacenes with increasing *n* was considered [31]:

(1) ΔE(n)=E([n]helicene)−E([n]phenacene)

Figure 3b shows the dependence ∆*E*(*n*) based on the calculated data from [31] at the B3LYP/6-31G* level. The results show a linear increase in the Δ*E* between helicenes and phenacenes with increasing *n*. In other words, an increase in *n* does not lead to any stabilization of helicene. At *n* = 16 (largest experimentally synthesized helicene), the difference reaches 83.4 kcal/mol (the result of B3LYP/6-31G* calculations). This makes it possible to understand the observed difficulties in obtaining helicenes with a further increase in *n*.

The difference in magnetic susceptibilities between helicenes and phenacenes was also investigated in [31]. This quantity enables us to determine the aromaticity of the studied molecules. According to the data of [31], helical distortions in the π-system expectedly lead to a decrease in the aromaticity of helicenes in comparison with phenacenes. However, this decrease is not so large—the loss of aromaticity in helicenes is approximately 10–13% compared to phenacenes.

**Figure 4:**
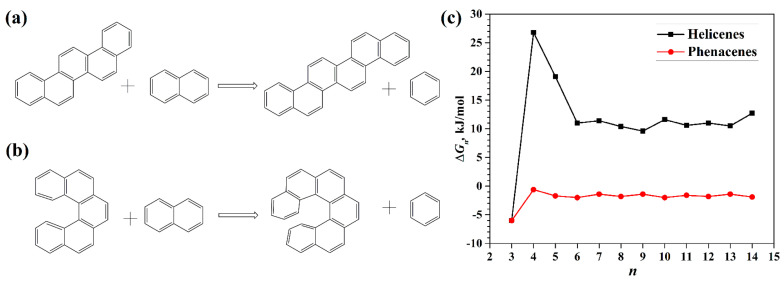
Examples of homodesmic addition of naphthalene to (**a**) phenacene and (**b**) helicene. (**c**) The Δ*G_n_* dependence of helicenes and phenacenes, according to data from Ref. [32].

Similar results were obtained by the authors of [32], who performed DFT calculations using the functional B3LYP with the TZVP basis set and the empirical Grimme correction to account for the dispersion forces [33] and applying the conductor-like screening model (COSMO) [34] to take into account the solvent (acetonitrile). Incremental Gibbs free energies, Δ*G_n_*, were obtained, which show the difference between the homodesmic addition of naphthalene (C_10_H_8_) to helicene or phenacene (see Figure 4a,b):

(2) $$\Delta G_{n}=G\left(\left[n\right]\right)+G\left(\mathrm{benzene}\right)-G\left(\left[n-1\right]\right)-G\left(\mathrm{naphthalene}\right)$$

Here, *G*([*n*]) and *G*([*n* − 1]) are the Gibbs free energies of [*n*]helicene or [*n*]phenacene and [*n* − 1]helicene or [*n* − 1]phenacene, respectively. According to the graph in Figure 4c, the Δ*G_n_* reaches a constant value of ~10 kJ/mol at *n* ~ 6 for helicenes, which qualitatively corresponds to the result of [31] on inherent instability and explains the difficulty of the synthesis of helicenes with a large *n*. A similar result was obtained at the M06-2X/6-311+G(3d,p) level of calculations [35].

We also note that the small difference between the aromaticity of helicenes and phenacenes noted earlier in [31] is confirmed by the results of subsequent works [32,35].

**Figure 5:**
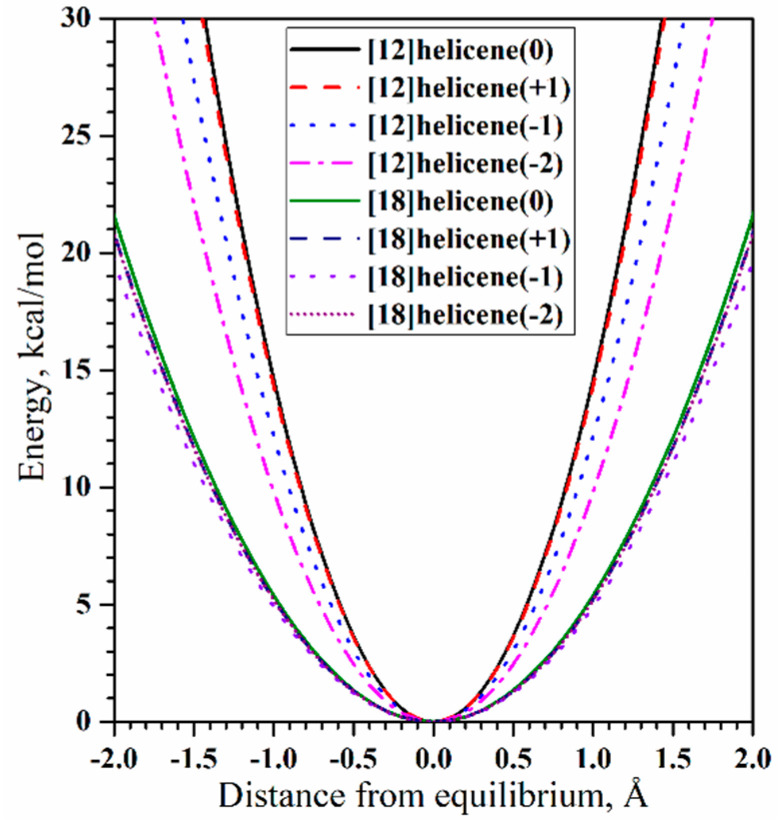
Energy dependence on the axial stress of the neutral, anionic, and cationic states of [12]helicene and [18]helicene, according to data from Ref. [36].

For the first time, the possibility of using helicene in terms of springs for application in nanotechnologies was discussed in [36]. From this point of view, at the level of the semi-empirical PM3 Hamiltonian, the force constants of axial tension for neutral helicenes and their cationic and anionic forms were estimated. Calculations were made for helicenes with *n* = 12 and 18, which corresponds to the number of coils of two and three, respectively (Figure 5). According to [36], the force constants are in the range 9.8–14.6 kcal·mol^−1^·Å^−2^ for small “springs” and approximately in the range 4.9–5.4 kcal·mol^−1^·Å^−2^ for large “springs”. The differences in hardness depend little on the electronic state of helicenes (i.e., neutral, cationic, or anionic states).

In [32], a somewhat larger value was obtained for [14]helicene, equal to 23.6 kcal·mol^−1^·Å^−2^. It can be assumed that the difference is due to a more accurate calculation method, which was carried out within the DFT (B3LYP functional) in the TZVP basis set, taking into account the solvent and dispersion corrections. A further theoretical study of the influence of the axial deformations of [∞]helicene and its non-hexagonal modifications on mechanical stability, deformability, and fracture processes was carried out in [37] at the PBE/PAW level of theory. It was found that the presence of pentagons or heptagons instead of hexagons had a significant influence on the mechanical properties, and some helicenes could achieve very large reversible tensile strains (more than 200%). The roughly estimated effective tensile strength of pristine [∞]helicene is equal to 117 GPa.

The authors of [32] also made an attempt to computationally evaluate the convergence of the physicochemical properties of [*n*]helicenes with increasing *n*. The results of TD DFT calculations of excitation energies [32] agree well with experiments and demonstrate the convergence of the results at *n* ~ 14. In particular, for [14]helicene, the calculated wavelength for the *S*_0_ → *S*_1_ vertical transition is 476 nm (2.60 eV).

The convergence of the gap between the HOMO (highest occupied molecular orbital) and LUMO (lowest unoccupied molecular orbital) was studied at the B3LYP/6-31G* computational level [38]. Calculations were carried out for helicenes and phenacenes (and their analogs with sulfur) with an *n* of up to 30 and under PBCs (periodic boundary conditions) in order to obtain the change in the electronic band gap of the structures with *n* → ∞. The data obtained for [14]helicene in [38] correlate with the results in [32], taking into account the fact that the HOMO–LUMO gap is only an upper estimate of the energies of the *S*_0_ → *S*_1_ vertical transition obtained by the TD DFT.

**Figure 6:**
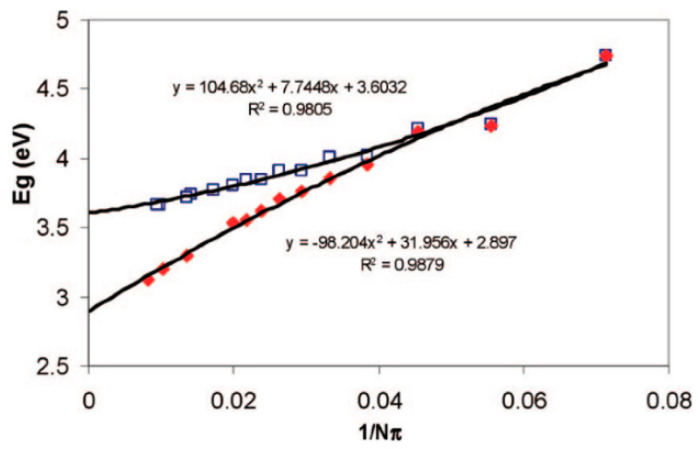
HOMO-LUMO gaps, Eg, for phenacenes (squares) and helicenes (diamonds) as a function of 1/Nπ. Nπ is the number of π electrons. Quadratic fits and the corresponding statistics are also shown. *n* of up to 26 and 30 were used for the phenacenes and helicenes, respectively. Reprinted with permission from Ref. [38] (Copyright 2008 American Chemical Society).

The graphs in Figure 6 demonstrate the difference between the HOMO–LUMO gap dependences on the π-electron numbers for [*n*]helicenes and [*n*]phenacenes and enables one to obtain the band gap values by extrapolation for *n* → ∞. The obtained extrapolations are very close to the results of the calculations under PBCs (2.90 eV and 3.59 eV for [∞]helicene and [∞]phenacene, respectively [38]). The value 2.90 eV correlates with the 2.49 eV value obtained for [∞]helicene by the authors of earlier work [39].

**Figure 7:**
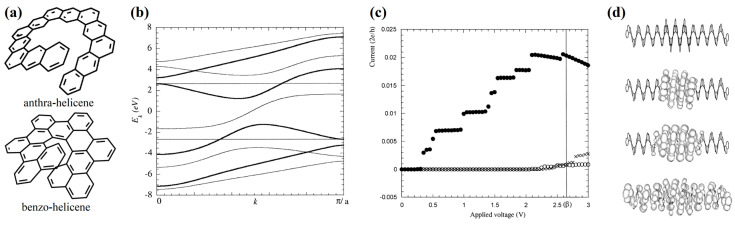
(**a**) The structures of anthra-helicene and benzo-helicene according to Ref. [40]. (**b**) The electronic bands for a semi-conductor [∞]helicene (thick curve) and a metallic [∞]benzo-helicene (thin curve); (**c**) *I*/*V* characteristics for the metallic benzo-helicene (filled circle), the semi-conductor helicene (open circle) and the hybrid metal–semi-conductor helicene (cross); (**d**) the structure and the molecular orbitals of a quantum dot realized by a semiconductor–metal–semiconductor helicene hybrid. From top to bottom: atomic structure; the MO at Fermi level; successive localized states are encountered; the MO at the band edge of the semiconductor part. (**b**–**d**) reprinted from Ref. [39] (Copyright 1999, with permission from Elsevier).

To the best of our knowledge, Ref. [39] is the first work in which the infinite [∞]helicene was also considered (in the periodic model at the tight-binding level). In addition, we note that in [39], not only simple [∞]helicene but also its other topological variants (anthra-helicene and benzo-helicene) were considered for the first time (Figure 7a). [∞]benzo-helicene has been shown to be potentially metallic, with a zero band gap, unlike simple [∞]helicene, which is a semiconductor.

The band structure of these two 1D structures shows that the [∞]benzo-helicene conduction band is “inside” the band gap of [∞]helicene (Figure 7b), which allowed the authors of Ref. [39] to propose a variant of a quantum dot constructed as a hybrid of semi-conductor helicene and metallic benzo-helicene. The *I*/*V* characteristics (i.e., current-voltage) were calculated for finite size [21]benzo-helicene, [24]helicene, and their hybrid. As expected from the band structure, the *I*/*V* curve of the hybrid is similar to that of semi-conductor helicene (Figure 7c). This can also be understood in terms of the molecular orbitals (MO) of the hybrid. It can be seen from the figure that the MOs, which correspond to the energy levels within the band gap of the semiconductor part, will be localized on the metal part of the hybrid. If the voltage exceeds the band gap value of the semiconductor part, then the conductivity is restored, and MOs are delocalized throughout the hybrid (Figure 7d). To the best of our knowledge, hybrids that combine different types of helicenes have not been theoretically studied anywhere except in [39].

We also note that in [39], for the first time, such issues as the Peierls distortion of metallic helicene were raised, and a decrease in the band gap with “expanding” helicene (i.e., a transition from simple helicene to anthra-helicene) was also shown. These issues were repeatedly analyzed in subsequent theoretical works on nanohelicenes.

**Figure 8:**
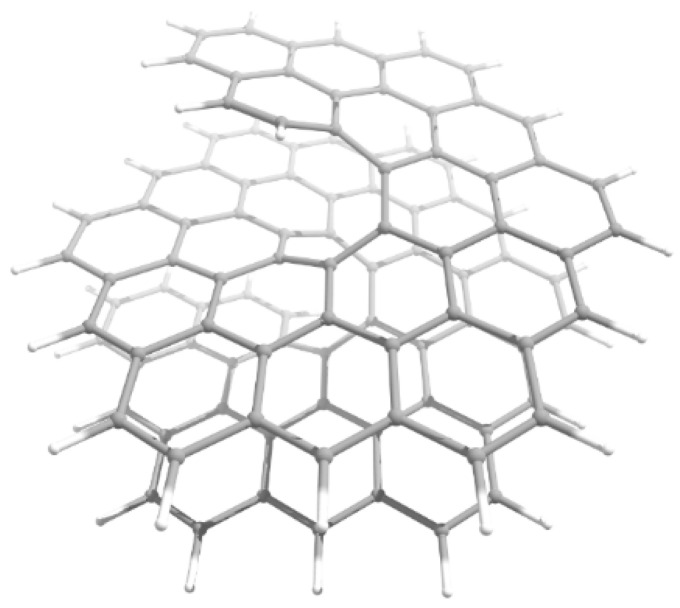
The atomic structure of the [12]helicene with a laterally-extended π-system, according to Ref. [40]. The gray and white spheres are carbon and hydrogen atoms, respectively.

In [40], the stability and properties of some other classes of helicenes were theoretically studied at the B3LYP/6-31G* level. Some ways for constructing a spiral from various combinations of hexagons, pentagons, and tetragons from carbon atoms were considered, including the “lateral-extending” of the helicene’s π-system (Figure 8), which is the object of study in subsequent theoretical works.

The presence of a continuous π-system and axial chirality makes helicenes and their derivatives promising for achieving non-linear optical (NLO) properties. At the level of the semi-empirical AM1 Hamiltonian, within the framework of the time-dependent Hartree-Fock (TD HF) theory, the change in the first hyperpolarizability ($\bar{\beta }$) and its projection on the dipole moment ($\beta_{∥}$) were theoretically studied with a successive increase in *n* (up to *n* = 19) for helicenes and their antiaromatic analogs, phenylenes [41]. It was found that $\bar{\beta }$ increases monotonically with the size of the system. At the same time, in helicenes, $\beta_{∥}$ is positive and presents quasi-periodic oscillations with the helix.

**Figure 9:**
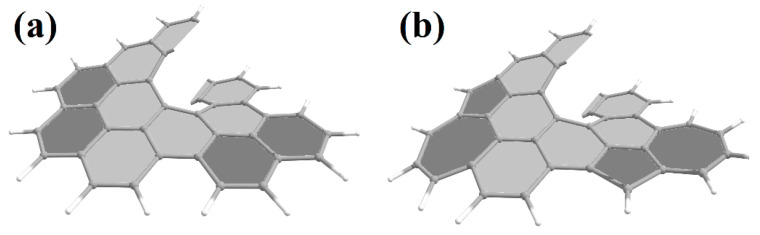
The [∞]helicenes with the π-system extended by (**a**) naphthalene or (**b**) azulene units, respectively, according to Ref. [42]. The gray and white spheres are carbon and hydrogen atoms, respectively.

It was theoretically shown at the PBEPBE/6-31G(d,p) level that the modification of the helicene backbone by fusing the azulene element (Figure 9) leads to a significant enhancement of the second-order NLO properties due to the polar charge distribution in the pentagon end (a component of azulene) [42]. NLO properties have also been shown to exhibit only slight changes under an external strain.

Further works in this area concerned third-order NLO properties, two-photon circular dichroism, and so on. A detailed review of the calculations and experiments on the NLO properties of helicenes and their derivatives is presented in a recent book [43].

Theoretical studies of electron transport through helicenes were the subject of works [44,45,46,47,48]. The common result for all the works mentioned is the conclusion about the presence of a strong dependence of conductivity and other properties on the applied mechanical stress. This is a fairly expected result based on the understanding of helicenes as molecular springs.

**Figure 10:**
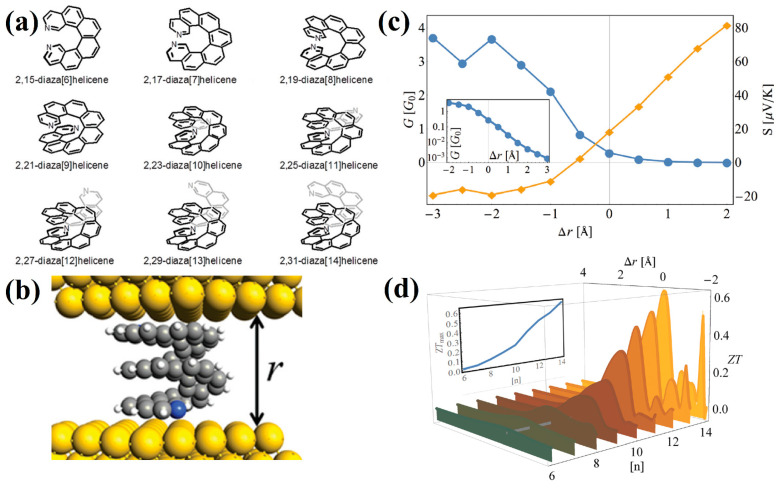
(**a**) Chemical structure of diaza[*n*]helicenes used in the DFT and DFTB calculations; (**b**) 2,31-diaza[14]helicene molecule connected to two Au(111) contacts; *r* denotes the distance between the electrodes; (**c**) 2,21-diaza[9]helicene and Au(111) electrodes: conductance *G* (blue circles) and thermopower *S* (yellow diamonds) as a function of Δ*r* (inset: conductance on a logarithmic scale); (**d**) *zT* as a function of Δ*r* for a series of homologous diaza[*n*]helicenes (inset: maximal thermoelectric FOM, *zT_max_*, as a function of *n*). Adapted from Ref. [44].

In particular, helicene-based molecular junctions constructed as (diaza)helicenes placed between two Au(111) electrodes were studied in [44] (Figure 10a,b). Helicene compression leads to a significant increase in conductance, whereas stretching leads to a decrease. In the range of low voltages, conductance falls off linearly on a logarithmic scale as a function of strain (Figure 10c). The thermopower shows a sign change, which implies a change in the transport mechanism of the junction. The dependence of the thermoelectric *figure of merit* (FOM), *zT*, on strain for increasing *n* values shows that the maximal values of FOM were obtained for the longest helicene (Figure 10d). The maximal *zT* values can be as large as ~0.6, which far exceeds the values for currently known molecular junctions.

**Figure 11:**
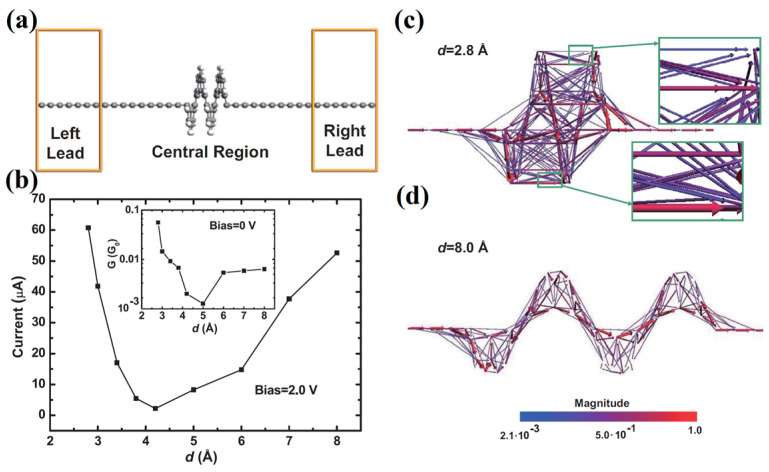
(**a**) The setup of the two-probe system of carbon chain–[12]helicene–carbon chain; (**b**) current dependence on the distance between coils *d* under the bias of 2.0 V (inset: conductance under zero bias on a logarithmic scale); transmission pathways (**c**) for *d* = 2.8 Å case and (**d**) for *d* = 8.0 Å case (the volume and color of the arrow illustrate the magnitude of the pathway). Adapted from Ref. [45].

In [45], a wider tension-compression interval for [12]helicene (Figure 11a) was theoretically investigated, and the results on *I/V* behavior correlate with the results of [44] in the corresponding interval. However, the use of a wider interval made it possible to detect an increase in conductivity in the region of large stretches. Thus, current dependence on pitch has a *U*-shape (Figure 11b). The first part of the curve (the distance between coils is less than 4 Å) corresponds to the transmission pathways between helicene coils (through space) (see Figure 11c). The ascending part of the curve corresponds to a change in the conduction mechanism, and the conduction is due to transmission along the helicene ribbon (through bond) (see Figure 11d).

At PBE/DZP computational level, the authors of [46] investigated the electronic transport properties of spiral-shaped molecules coupled to metal electrodes. Three molecules were composed of [7]helicene and two thiol-[7]helicenes with benzenethiolates on both sides. The data on local transmission pathways demonstrate that there is no spiral current flowing through [7]helicene, and the total current is only due to electron tunneling in the inter-layer. In the case of thiol-[7]helicenes, the electron distribution in frontier orbitals will change, and the spiral current will appear, which makes it possible to generate a magnetic field. The negative differential resistance behavior of *I*/*V* curves was also found in [46]. In other words, in a certain interval of bias voltage, the current decreases with bias increases.

A similar result about the absence of a helical current for helicenes was obtained in [47]. It was also shown in [48] that the conductivity of the oligomers of [*n*]benzo-helicene (see [39]) is significantly higher than that of conventional [*n*]helicenes. In the limit, this corresponds to the metallic conductivity of [∞]benzo-helicene that was shown in [39].

In addition to electrical conductivity studies, the theoretical calculations were also concerned with the potential application of helicene molecules to control spin transport. Such control has many advantages over conventional electronic transport and can potentially be used in nanodevices that are smaller and more robust than modern nanodevices. The use of helicene derivatives as a spin filter exhibiting the CISS effect was shown experimentally [49]. According to the general theory, the driving force behind CISS is spin-orbit interaction, SOC. Despite the fact that for carbon atoms, the SOC value is very small (about 6 meV), the CISS for these molecules can be significant [50], which opens up great opportunities for the use of organic chiral molecules, and in particular, helicenes.

The first theoretical consideration of spin-polarized electron transport in helicene was carried out in [51]. The [*n*]helicenes (*n* = 12, 16, 20, and 24) combined with semi-infinite graphene nanoribbons were considered. The spin polarization, *P_s_*, is determined through spin-up *G*_↑_ and spin-down *G*_↓_ conductance, according to Equation (3):

(3) $$P_{s}=\left(G_{↑}-G_{↓}\right)/\left(G_{↑}+G_{↓}\right)$$

**Figure 12:**
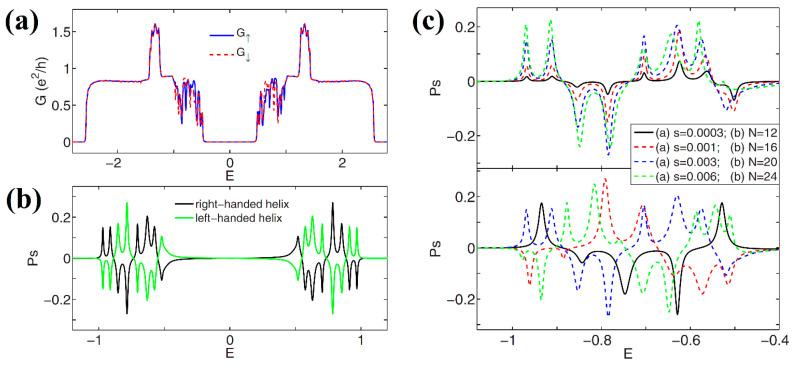
(**a**) Energy-dependent spin-up *G*_↑_ and spin-down *G*_↓_ conductance for the right-handed [20]helicene; (**b**) spin polarization *P_s_* vs. Fermi energy *E* for the right-handed and left-handed [20]helicene molecules; (**c**) *P_s_*(*E*) curves with various spin-orbit coupling strength, *s* (top panel), and *P_s_*(*E*) for [*N*]helicenes with various *N* (bottom panel). Reprinted with permission from Ref. [51] (Copyright 2016 American Physical Society).

Using the tight-binding Hamiltonian with the addition of the SOC Hamiltonian, it was shown that the *P_s_* of [20]helicene could reach 27%, which is comparable to the experimentally found values in [49]. Interestingly, *P_s_* exhibits significant oscillations by varying the Fermi energy, *E*, because of quantum interference effects (Figure 12a). In addition, the influence of axial deformations on *P_s_* was studied. Axial deformations significantly change the overall shape of the *P_s_*(*E*) curve; however, the oscillation behavior remains in the energy spectrum regardless of the applied mechanical stress, and the spin filtration efficiency remains quite large (Figure 12b). The *P_s_*(*E*) curves for other helicenes differ significantly in terms of peak heights and their location, but the overall picture remains the same as in the case of [20]helicene (Figure 12c).

Since the CISS effect relates primarily to excited electron states, it should be observed not only in the case of flowing current but also in the case of a photocurrent promoted by photo-excited electrons. In [52], an experimental and theoretical study of [7]helicene molecules adsorbed on metal surfaces (Cu(332), Ag(110), and Au(111)) was carried out. An excess of 6–8% of longitudinal spin polarization, *P*_Z_, was demonstrated:

(4) $$P_{Z}=\frac{I_{↑}-I_{↓}}{I_{↑}+I_{↓}}S_{\mathrm{eff}}^{-1}$$

**Figure 13:**
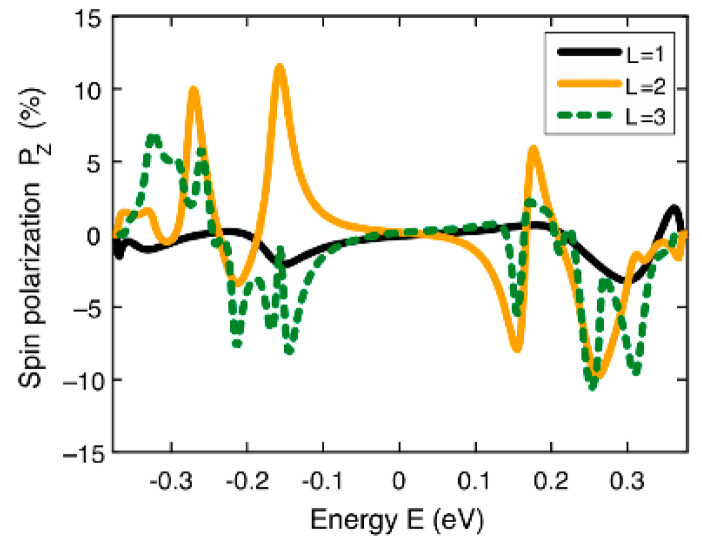
The *P_Z_*(*E*) dependences for the model helical system computed for different numbers of left-handed helical coils, L. Reprinted from Ref. [52].

Here, *I*_↑,↓_ denotes the count rates in the two detectors, and *S*_eff_ is the effective Sherman function. It is important to note that circularly polarized UV/Vis light served as the source of spin polarization. Thus, the obtained experimental results connect two promising phenomena—CISS and CPL, which is possible in this case. In the computational part of Ref. [52], the DFT method was used to study the [7]helicene molecules adsorbed on different surfaces. Then, using the model Hamiltonian to calculate the CISS, *P_Z_* was obtained for helicenes with different numbers of coils (Figure 13). The results showed a significant increase in *P_Z_* for the system with the maximum number of coils (three in the case under study). Interestingly, the authors of [53] explored the CISS associated with inter-system crossing (ISC) in carbon-sulfur [*n*]helicenes. It has been suggested that the efficient ISC of these molecules might be beneficial for an efficient CISS.

**Figure 14:**
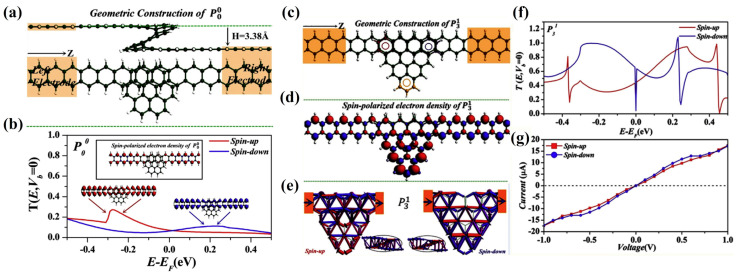
Standard helicene: (**a**) the geometry of a two-probe construction; (**b**) the equilibrium spin-dependent transmission *T*(*E*) with the spin-polarized local electron density of states around the selected peaks and the electronic spin density (inset). Trigonal modification of helicene: (**c**) the geometry of two-probe construction; (**d**) the electronic spin density; (**e**) the spin-dependent local transmission pathways (the green arrows represent the transport direction); (**f**) the equilibrium spin-dependent transmission *T*(*E*); (**g**) the spin-dependent *I*/*V* curve. Reprinted from Ref. [54] (Copyright 2017, with permission from Elsevier).

Spin transport through helicenes bonded to a carbon zigzag-terminated nanoribbon was theoretically studied in [54] (Figure 14). Some peripheral modifications of helicene with one coil were considered, starting from the basic [6]helicene. The helicene periphery was successively expanded by adding from one to three carbon hexagons. The calculations were carried out at the DFT+NEGF (non-equilibrium Green function) level with the DZP basis set.

It was shown that in the case of basic helicene (Figure 14a), both spin states on the central spiral moiety reveal transport via inter-layer tunneling without the generation of spiral current, which is similar to the results of [45,46]. Spin-polarized transport is present but is relatively small (Figure 14b). However, modification by adding carbon hexagons resulted in a robust spin-polarized interaction, especially in the case of the appearance of trigonal sections (Figure 14c,f,g). According to the Lieb theorem [55] (regarding the total spin of the Hubbard model in bipartite sublattices *A* and *B*), these parts have extra atoms that break the balance between the number of atoms in sublattices, i.e., *N_A_* ≠ *N_B_*. Consequently, the emergence of magnetism is induced by edge imbalance (Figure 14d). Additionally, depending on the location of additional hexagons, helicene-based devices exhibit an on-off spin-polarization phenomenon, which manifests itself in the presence/absence of spin-splitting that is dependent on bias. Interestingly, the location of additional hexagons also affects the type of electron tunneling. In the case of trigonal modification, it was possible to redirect the current completely in a spiral, i.e., intra-layer tunneling is a major contribution to current, which is in contrast to the basic helicene case (Figure 14e).

Despite the fact that the theoretical works considered in this section mainly refer to molecular helicenes, the manifestation of many of the effects found can also be expected for nanohelicenes. We especially note the importance of structural modification, which consists of the addition of carbon hexagons to the carbon skeleton of the initial [*n*]helicene. From the results of theoretical studies, it can be clearly seen that modification leads to a significant change in the optical, electronic, and magnetic properties, and taking into account the spring topology of helicenes makes such modifications extremely promising for creating corresponding opto-, electro-, and magneto-mechanical devices at the nano level.

For a better understanding of the effects of the peripheral modification of the edges on the other properties, it is convenient to introduce a nomenclature of nanohelicenes. Based on the symmetry of graphene, the nomenclature distinguishes prototypical classes and makes it possible to consider all nanohelicenes as some peripheral modification of these classes.

## 3. Nomenclature of Nanohelicenes

The nomenclature presented here is based on the consideration of nanohelicenes as stacks of graphene nanoflakes or nanorings [16], to which the idea of a Riemann surface [56] is applied. Namely, each nanoflake is hypothetically cut up to the center, and one edge is stitched to the edge of the neighboring upper nanoflake, with the second one sewn to the neighboring lower nanoflake. The saturation of the uncompensated σ-bonds of graphene nanoflakes and nanorings with hydrogen atoms results in polyaromatic hydrocarbons (PAHs). More precisely, nanohelicenes are obtained by applying the idea of a Riemann surface to PAHs. Note that the π-system of the graphene nanoflake or nanoring and the corresponding PAHs is the same, which means that, according to the Lieb theorem [55], they will have qualitatively close properties, although, of course, ab initio calculations will give quantitatively different results since they take into account all the electrons.

This method of the construction of nanohelicenes makes it possible to use the theoretical results obtained for carbon nanoflakes (for example, see [57,58,59]) and leads to a large number of different classes of nanohelicenes. All of them, according to the molecular nomenclature of helicenes, will be [∞]helicenes, i.e., the number of carbon hexagons is *n* → ∞, so other parameters should be used. It is convenient to use parameters based on the symmetry of nanoflakes or nanorings from which nanohelicene is built.

**Figure 15:**
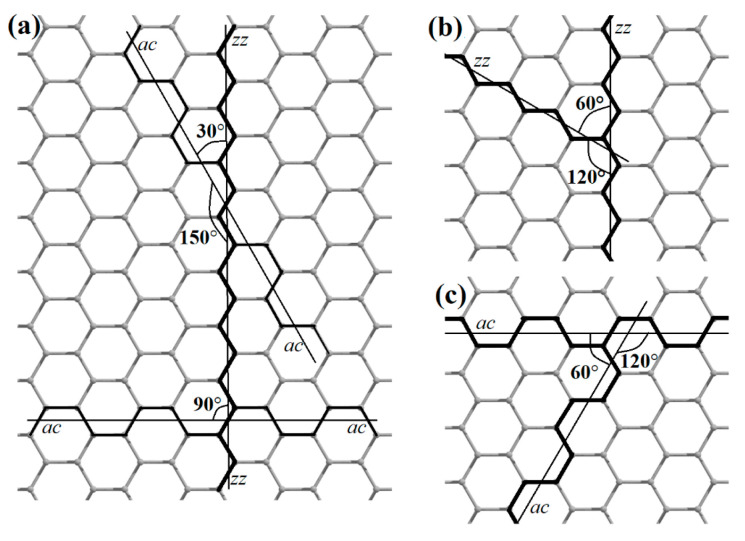
(**a**) The crossing between (*zz*) and (*ac*) cutting lines in a graphene sheet. The crossing between possible (**b**) (*zz*) and (**c**) (*ac*) cutting lines in a graphene sheet.

As is known, the most symmetrical ways of cutting a graphene nanoribbon are zigzag (*zz*) and armchair (*ac*). The cutting lines of these two types are located at an angle of 30°, 90°, or 150° relative to each other (Figure 15).

**Figure 16:**
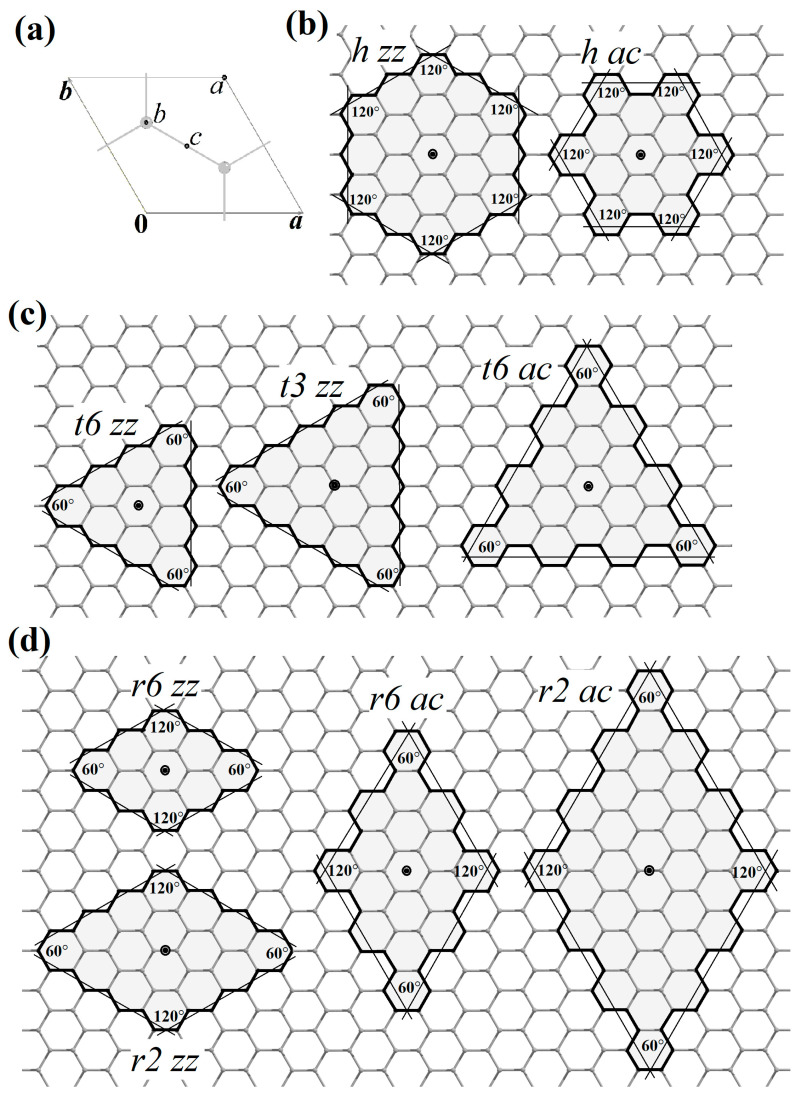
(**a**) Wyckoff positions *a*, *b*, and *c* in a graphene sheet; (**b**) hexagonal, (**c**) trigonal, and (**d**) rhombic variants of graphene nanoflakes. The bold black lines and double dots show the edges and the centers of the nanoflakes, respectively.

The cutting lines of the same type can be either at 120° or 60° relative to each other. If graphene is sequentially cut through the same number of hexagons at an angle of 120°, then the final object will be a graphene nanoflake of hexagonal shape (*h*) with symmetry indicated by the *D*_6h_ point group. If the graphene is cut at an angle of 60°, then a triangular shape (*t*) with *D*_3h_ symmetry is obtained. If the cut is made by alternating 120° and 60°, then a rhombic nanoflake (*r*) of *D*_2h_ symmetry (Figure 16) is obtained.

The *D*_6h_ symmetry of a hexagonal nanoflake or nanoring requires that the six-order axis passes through the center of the hexagon, which corresponds to the Wyckoff position *a* (Figure 16a) of the graphene symmetry group (*P*6/*mmm*, No 80 [60]). In this case, both considered terminations, (*zz*) and (*ac*), are possible (Figure 16b).

The *D*_3h_ symmetry of a trigonal nanoflake or nanoring can be realized in two ways. The first way is connected to *D*_3h_ symmetry realization as a subgroup of *D*_6h_, and in this case, the three-order axis also passes through the center of the hexagon (Wyckoff position *a*), so this variant can be denoted as (*t*6). In this case, both considered terminations, (*zz*) and (*ac*), are possible (Figure 16c).

The second way of *D*_3h_ symmetry appearance is realized if the three-order axis passes through the Wyckoff position *b* (Figure 16a) of the graphene symmetry group (the position occupied by the carbon atoms). The highest order of the rotation axis in the site symmetry group of this position is three, so this option is denoted as (*t*3). The construction of a trigonal shape with such centering is only possible in the (*zz*) termination (Figure 16c).

The *D*_2h_ symmetry of a nanoflake or nanoring of rhombic shape is also possible in two ways. The first one is connected to a subgroup of *D*_6h_, and in this case, the main two-order axis also passes through the center of the hexagon (Wyckoff position *a*), so this variant is denoted as (*r*6). In this case, (*zz*) and (*ac*) terminations are possible (Figure 16d).

The second variant of *D*_2h_ symmetry is realized if the second-order axis passes through the Wyckoff position *d* (Figure 16a) of the graphene symmetry group. This position corresponds to the center of the C-C bond. The maximum order of the main axis is two, so this variant is denoted as (*r*2). The construction of a rhombic morphology with such centering is possible for both considered terminations, *zz* and *ac* (Figure 16d).

Thus, nine types of graphene nanoflakes and the corresponding PAHs are possible, taking into account the following:

(1) the type of termination (*zz* and *ac*);
(2) the angle of rotation between them (*h* for 60°, *t* for 120°, and *r* in the alternating case);
(3) the centering in different Wyckoff positions (the presence of an axis of sixth, third, and second orders).

These types are (Figure 16):

*h zz*, and *h ac*;

*t*6 *zz*, and *t*6 *ac*;

*t*3 *zz*;

*r*6 *zz*, and *r*6 *ac*;

*r*2 *zz*, and *r*2 *ac*.

**Figure 17:**
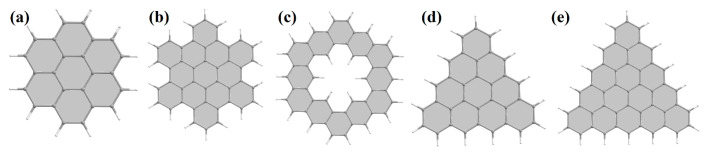
Examples of experimentally synthesized PAHs: (**a**) coronene; (**b**) hexa-peri-hexabenzocoronene; (**c**) kekulene; (**d**) [4]triangulene; (**e**) [5]triangulene. The gray and white spheres are carbon and hydrogen atoms, respectively.

Examples of experimentally synthesized PAHs of hexagonal shape are coronene and hexa-peri-hexabenzocoronene (Figure 17a,b). These molecules are examples of saturated graphene hexagonal nanoflakes with zigzag and armchair terminations, respectively. In the case of trigonal nanoflakes, the saturation of uncompensated σ-bonds with hydrogen atoms is not enough since, in this case, an uncompensated π-electron exists, and a radical is obtained. Radical triangular structures with zigzag terminations are called [*n*]triangulenes, where *n* is the number of hexagons on one side of the triangle. In Figure 17d,e, [4]triangulene and [5]triangulene are shown. The first of them corresponds to the (*t*6 *zz*) type of nanohelicenes, and the second to the (*t*3 *zz*) ones.

When cutting out the inner region according to the same rules that are followed for the outer edge, graphene nanorings or the corresponding PAHs will be obtained. An example of an experimentally synthesized PAH is the kekulene (Figure 17c), which corresponds to the smallest possible graphene ring.

The nanoflakes under consideration have a hexagonal, trigonal, or rhombic shape and belong to the *D*_6h_, *D*_3h_, or *D*_2h_ symmetry groups, respectively. A “stack” of nanoflakes will have the *P*6/mmm (No 73), *P*-6m2 (No 71), or *P*mmm (No 20) rod groups, respectively. Rod groups are periodic 1D groups with crystallographic axis orders (1,2,3,4, and 6). A list of rod groups can be found in [60,61]. When a nanohelicene is formed, the main symmetry axis becomes a sixth-, third-, or second-order helical axis, respectively. The planes of symmetry and the operation of inversion disappear, but the perpendicular axes of the second order, *U*, remain. As a result, the symmetry of nanohelicenes will be described by the rod symmetry groups *P*6_1_22 (No 63), *P*3_1_12 (No 47), and *P*222_1_ (No 14). Group *P*3_1_12 can also be described as *P*3_1_21 (second setting of No 47). Additionally, we note the subgroups of these groups, which contain only rotations around the helical axis. These are rod groups *P*6_1_ (No 54) and *P*3_1_ (No 43), *P*112_1_ (No 9).

By using the principle laid down in [12] for the nomenclature of hexagonal nanohelicenes with a zigzag termination of the outer and inner edges, two indices can be introduced that determine the radius *m* of the *shaft* (the cutout inner region) and the width *n* of the *ribbon* measured using the number of carbon hexagons. Thus, all possible nanohelicenes of (*h zz*) type were identified by the name [*m*.*n*]helicene.

The radius, *R* (which equals the length of the outer edge), of nanohelicene, i.e., the distance from the center to one of the corners, was defined simply as

(5) R=m+n

In this nomenclature, the basic [∞]helicene was designated as [1.1]helicene. An increase in the ribbon width *n* leads to the *lateral-extended* nano-helicenes, according to [62]. An increase in the radius of the shaft, *m*, corresponds to the *expanded* nano-helicenes [62].

**Figure 18:**
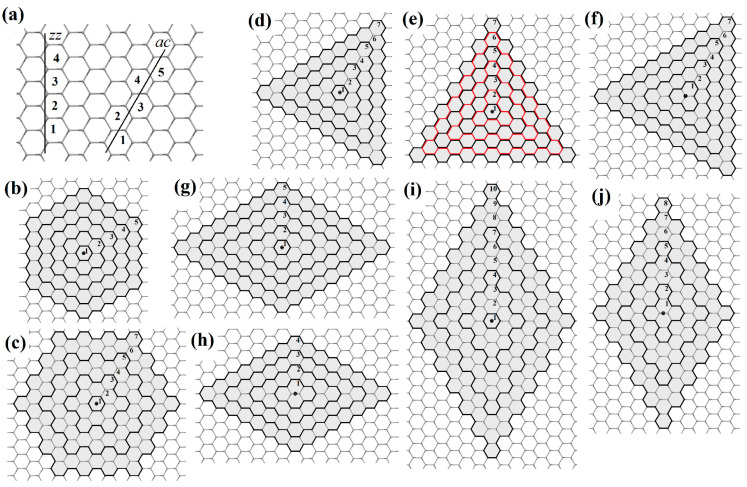
(**a**) Counting of *R* (Equation (5)) in the case of (*zz*) and (*ac*) terminations. Graphene nano-flakes of different morphologies and size: (**b**) *h zz*; (**c**) *h ac*; (**d**) *t*6 *zz*; (**e**) *t*6 *ac*; (**f**) *t*3 *zz*; (**g**) *r*6 *zz*; (**h**) *r*2 *zz*; (**i**) *r*6 *ac*; (**j**) *r*2 *ac*. The edge terminations are shown using bold black lines (in the case of (*t6 ac*) morphology the bold red line is used in addition to the black line). Methods for calculating *R* are shown for each considered morphology.

Next, we present a generalization of this nomenclature to other possible shapes and terminations discussed earlier for graphene nanoflakes and PAHs. To determine the indices *m* and *n*, Equation (5) is taken as basic because different terminations and shapes require slightly different ways of counting *R*. In Figure 18a, the ways of calculating the number of hexagons in the (*zz*) and (*ac*) directions are shown.

When the main axis is in the center of the hexagon (i.e., *h*, *t*6, *r*6 shapes), *R* includes half the hexagon and is rounded up to the nearest integer. For (*h*) shape, the way for counting *R* is determined by the type of edge termination (Figure 18b,c). For the (*t*6) shape, it is more appropriate to calculate *R* along with (*ac*) counting in the case of (*zz*) termination and vice versa (Figure 18d,e). When the main axis is centered on the atom (i.e., *t*3 shape), it is convenient to count the number of hexagons for *R* along with the (*ac*) direction (Figure 18f). Finally, in the cases of the (*r*6) and (*r*2) shapes, it is convenient to calculate the radius *R* in the (*zz*) direction for both possible terminations (Figure 18g–j).

Now we can determine the index *m*. By treating it as the radius of the shaft, we can obtain its value using the above rules. Finally, for a given *R* and *m*, the index *n* is determined by using Equation (5).

Such a definition of the indices *n* and *m* makes it possible to not only indicate nanohelicenes, in which the termination and shape of the shaft coincide with the termination and shape of the outer boundary, but also mixed variants. For example, in trigonal (*t*6) nanohelicene with (*ac*) external termination, a hexagonal shaft with (*zz*) inner termination can be cut. In order to take into account the possibility of the different morphology of the inner and outer terminations, additional upper and lower indices are introduced, which determine the shape and the termination, respectively. Thus, the nomenclature presented makes it possible to cover a large number of classes of nanohelicenes obtained by the enumeration of the shape indices (*h*, *t*6, *t*3, *r*6, and *r*2) and termination indices (*zz*, *ac*) of the numbers *m* and *n*.

Nano-helicenes, for which the shape and the edge termination are the same for the outer and inner boundaries, form nine classes:

[$m_{zz}^{h}.n_{zz}^{h}$]helicene;

[$m_{ac}^{h}.n_{ac}^{h}$]helicene;

[$m_{zz}^{t6}.n_{zz}^{t6}$]helicene;

[$m_{ac}^{t6}.n_{ac}^{t6}$]helicene;

[$m_{zz}^{t3}.n_{zz}^{t3}$]helicene;

[$m_{zz}^{r6}.n_{zz}^{r6}$]helicene;

[$m_{ac}^{r6}.n_{ac}^{r6}$]helicene;

[$m_{zz}^{r2}.n_{zz}^{r2}$]helicene;

[$m_{ac}^{r2}.n_{ac}^{r2}$]helicene.

Among nanohelicenes with different outer and inner morphology, we note those with an (*h*) shaft and (*zz*) termination. In such nanohelicenes, the steric hindrance is expected to be minimal:

[$m_{zz}^{h}.n_{ac}^{h}$]helicene;

[$m_{zz}^{h}.n_{zz}^{t6}$]helicene;

[$m_{zz}^{h}.n_{ac}^{t6}$]helicene;

[$m_{zz}^{h}.n_{zz}^{r6}$]helicene;

[$m_{zz}^{h}.n_{ac}^{r6}$]helicene.

**Figure 19:**
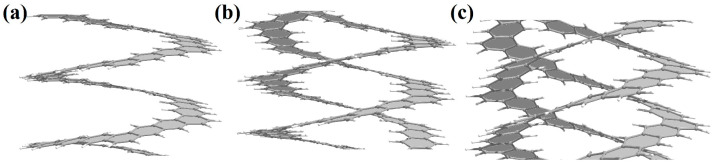
The atomic structures of (**a**) mono[$3_{zz}^{h}.1_{zz}^{h}$]helicene; (**b**) di[$3_{zz}^{h}.1_{zz}^{h}$]helicene; (**c**) tri[$3_{zz}^{h}.1_{zz}^{h}$]helicene. The gray and white spheres are carbon and hydrogen atoms, respectively.

Nanohelicenes consisting of two or three helices can also be easily obtained using the same construction principles. The only difference will be that instead of one cut followed by stitching, there should be two or three cuts for a double or triple helix, respectively. Since the structure of the graphene nanoflakes for single, double, or triple helices is the same, the prefix “di” or “tri” will simply be added to the corresponding name of nanohelicene. The representative examples can be seen in Figure 19.

**Figure 20:**
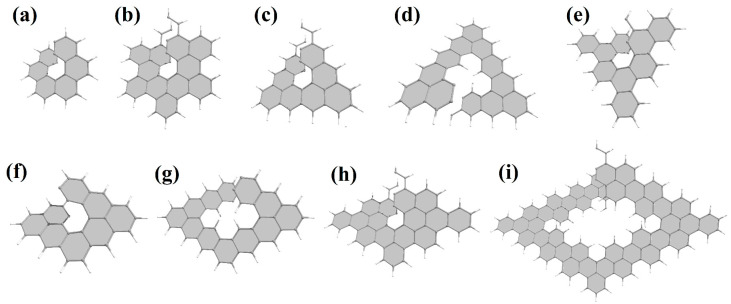
Atomic structures (one coil) of nanohelicenes with minimal shafts and ribbons. (**a**) [$1_{zz}^{h}.1_{zz}^{h}$]helicene; (**b**) [$1_{ac}^{h}.2_{ac}^{h}$]helicene; (**c**) [$1_{zz}^{t6}.2_{zz}^{t6}$]helicene; (**d**) [$1_{zz}^{t3}.2_{zz}^{t3}$]helicene; (**e**) [$1_{ac}^{t6}.2_{ac}^{t6}$]helicene; (**f**) [$1_{zz}^{r6}.1_{zz}^{r6}$]helicene; (**g**) [$1_{zz}^{r2}.1_{zz}^{r2}$]helicene; (**h**) [$1_{ac}^{r6}.3_{ac}^{r6}$]helicene; (**i**) [$2_{ac}^{r2}.3_{ac}^{r2}$]helicene. The gray and white spheres are carbon and hydrogen atoms, respectively.

**Figure 21:**
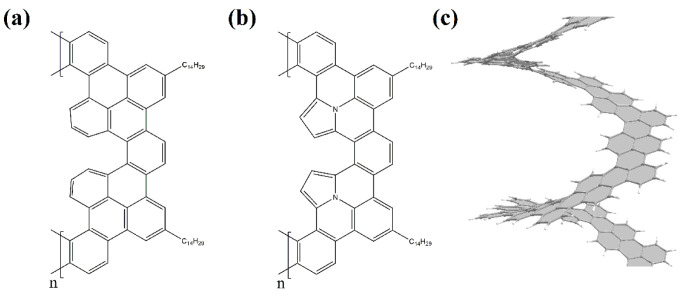
Synthesized oligomers of hexagonal *ac*-terminated nanohelicenes with inner edge modification: (**a**) benzene-embedded, according to Ref. [63]; (**b**) pyrrole-embedded, according to Ref. [64]. (**c**) The structure of prototypical [$3_{ac}^{h}.2_{ac}^{h}$]helicene. The gray and white spheres are carbon and hydrogen atoms, respectively.

Finally, let us consider in more detail the morphology of nanohelicenes with minimal shafts and ribbons (see Figure 20). In the basic case of [∞]helicene (i.e., [$1_{zz}^{h}.1_{zz}^{h}$]helicene in the proposed nomenclature), the shaft has an (*h*) shape with a size of one hexagon (Figure 21a). The same shaft with *m* = 1 will exist in the cases of the (*t*6) and (*r*6) shapes of both possible terminations (Figure 20a–c,e,f,h).

In the case of the (*t*3) shape, the minimal shaft, *m* = 1, will already consist of three hexagons having a common vertex and will have a triangular shape (Figure 20d). In the case of the (*r*2) shape and (*zz*) termination, the minimal shaft, *m* = 1, will consist of four hexagons forming a rhombus (Figure 20g). Finally, in the case of nanohelicenes with (*r*2 *ac*) morphology, the minimal shaft consists of six hexagons with *m* = 2 (Figure 20h).

To date, a large number of nanohelicenes have been theoretically considered, which differ in their design of the inner and outer edges. Nevertheless, all of them can be represented as a modification of the periphery of the ribbon of nanohelicenes when using the nomenclature presented here.

We also note that this nomenclature is based on graphene and, therefore, does not describe variants where the ribbon is formed not only by hexagons but also by pentagons or squares, as well as nanohelicenes with hetero-atoms.

## 4. Current State of Experimental Possibilities for the Synthesis of Nano-Helicenes

In this section, we briefly mention the experimental studies devoted to two directions: (i) the preparation of nanohelicenes with a large number of coils and (ii) the synthesis of helicenes with an edge termination and ribbon width different to that of conventional [*n*]helicenes.

As already mentioned, increasing the number of benzene rings in molecular helicenes is a very difficult experimental problem, and at present, only 16 rings have been connected, forming [16]helicene (see [23]). According to the theoretical calculations considered earlier [31,32,35], this is due to steric hindrances, which are maximal for the atoms closest to the helical axis. Thus, it can be expected that obtaining infinite [$1_{zz}^{h}.1_{zz}^{h}$]helicene is problematic.

Currently, experimental groups working on the synthesis of nanohelicenes are trying to avoid these steric hindrances, in particular, by synthesizing expanded nanohelicenes (i.e., *m* > 1). In more detail, it was possible to synthesize expanded nanohelicene, which contains about 32 monomers or five coils (assuming that there are six monomers in a coil) [63]. According to the presented nomenclature, this is an oligomer of hexagonal-shaped nanohelicene (Figure 21a). The outer edge of the ribbon has an (*ac*) termination, and the inner edge is some peripheral modification of the (*ac*) termination, in which one of the hexagons is “shifted” from one place to another. In a recent work by this group [64], a pyrrol-embedded variant was synthesized (approximately 34 monomers), where a pyrrole pentagon was used instead of a benzene hexagon in some places on the periphery of the inner edge (Figure 21b). The closest morphology to these mentioned nanohelicenes corresponds to the [$3_{ac}^{h}.2_{ac}^{h}$]helicene (Figure 21c).

**Figure 22:**
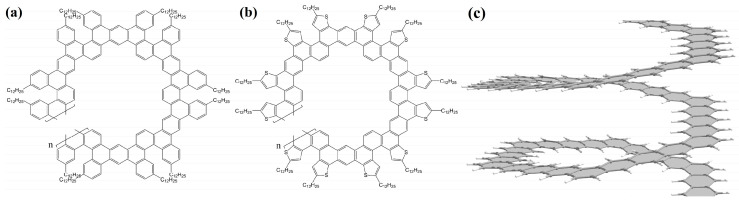
Synthesized oligomers of hexagonal *zz*-terminated nanohelicenes, according to Ref. [45]: (**a**) benzene-modified and (**b**) thiophene-modified outer edges. (**c**) Structure of prototypical [$4_{zz}^{h}.1_{zz}^{h}$]helicene. Gray and white spheres are carbon and hydrogen atoms, respectively.

The same group synthesized nanohelicenes, the carbon skeleton of which can be defined as a modification of [$4_{zz}^{h}.1_{zz}^{h}$]helicene [65] (Figure 22). Here, we can also note the wide possibilities of edge design due to the replacement of outer hexagons with pentagons (in this case, thiophene). Thus, the works [63,64,65] demonstrate the fundamental possibility of synthesizing polymers with an expanded nanohelicene skeleton.

**Figure 23:**
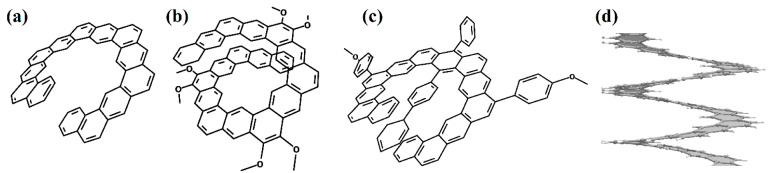
Synthesized oligomers of hexagonal *zz*-terminated nanohelicenes, according to (**a**) Ref. [66]; (**b**) Ref. [67]; (**c**) Ref. [68]. (**d**) Structure of prototypical [$2_{zz}^{h}.1_{zz}^{h}$]helicene. The gray and white spheres are carbon and hydrogen atoms, respectively.

In [66], one coil of pure [$2_{zz}^{h}.1_{zz}^{h}$]helicene was synthesized (Figure 23a). This nanohelicene is quite convenient for synthesis from a practical point of view, and the authors of [67] presented further progress in increasing the length of the ribbon and reaching a second coil of up to 17 carbon hexagons (Figure 23b). The authors of [68] also synthesized one coil of [$2_{zz}^{h}.1_{zz}^{h}$]helicene with internally incorporated phenyl- and biphenyl-substituents (Figure 23c).

**Figure 24:**
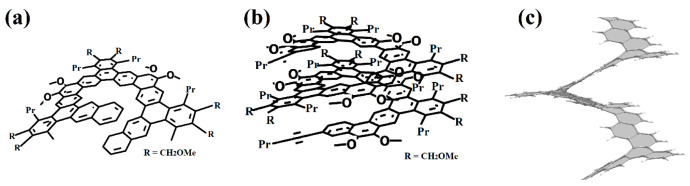
Synthesized oligomers of trigonal armchair-terminated nanohelicenes, according to (**a**) Ref. [62]; (**b**) Ref. [69]. (**c**) Structure of prototypical [$2_{ac}^{t6}.2_{ac}^{t6}$]helicene. The gray and white spheres are carbon and hydrogen atoms, respectively.

In [62], one coil was synthesized (Figure 24a), and in [69], two coils of nanohelicene were synthesized (Figure 24b), which can be considered a modification of [$2_{zz}^{h}.1_{zz}^{h}$]helicene. Note that it is also possible to consider this structure as a modification of [$2_{ac}^{t6}.2_{ac}^{t6}$]helicene (Figure 24c), which may be useful for future synthetic approaches.

**Figure 25:**
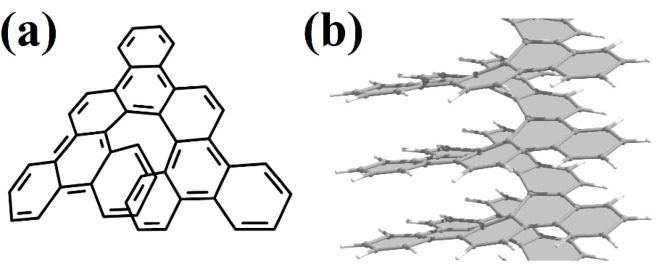
(**a**) Synthesized oligomer of pristine [$1_{ac}^{t6}.2_{ac}^{t6}$]helicene, according to Ref. [70]. (**b**) Structure of prototypical [$1_{ac}^{t6}.2_{ac}^{t6}$]helicene. The gray and white spheres are carbon and hydrogen atoms, respectively.

One coil of pure [$1_{ac}^{t6}.2_{ac}^{t6}$]helicene was synthesized in [70] (Figure 25a). This structure is the first example of helicene with an (*ac*) termination at the edges. 

**Figure 26:**
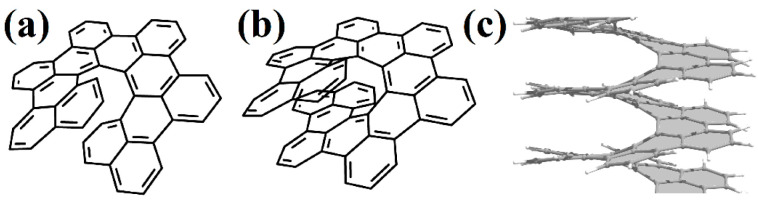
Synthesized oligomers of pristine [$1_{ac}^{h}.2_{ac}^{h}$]helicene, according to (**a**) Ref. [71]; (**b**) Ref. [72]. (**c**) Structure of prototypical [$1_{ac}^{h}.2_{ac}^{h}$]helicene. The gray and white spheres are carbon and hydrogen atoms, respectively.

Next, armchair-terminated helicene was synthesized in [71] (Figure 26a), which can be considered as one coil of [$1_{ac}^{h}.2_{ac}^{h}$]helicene. The second coil of this nanohelicene was “started” in [72] (Figure 26b).

**Figure 27:**
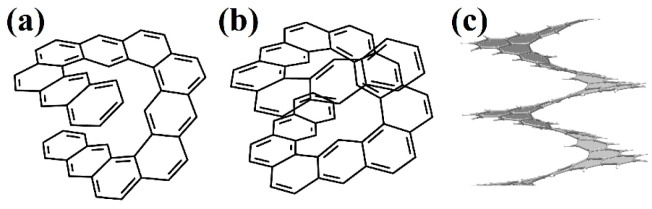
Synthesized oligomers of modified trigonal (*t*3) *zz*-terminated nanohelicenes, according to Ref. [73]: (**a**) with one coil and (**b**) with one and a half coils. (**c**) Structure of prototypical [$1_{zz}^{t3}.2_{zz}^{t3}$]helicene. The gray and white spheres are carbon and hydrogen atoms, respectively.

One and a half coils of nanohelicene were synthesized in [73] (Figure 27a), which can be considered as a modification of [$1_{zz}^{t3}.2_{zz}^{t3}$]helicene (Figure 27b) with the external corner hexagons cut out.

**Figure 28:**
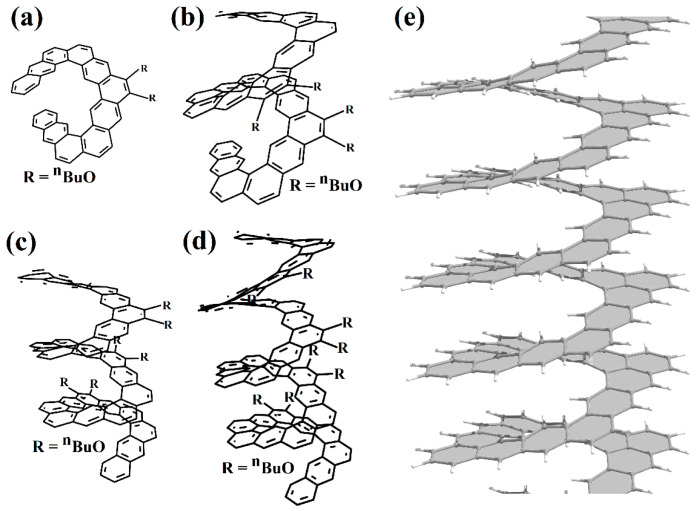
(**a**) one, (**b**) two, (**c**) three, and (**d**) four coils of the modification of rhombic nanohelicene according to Ref. [74]. (**e**) Structure of prototypical [$1_{zz}^{r2}.2_{zz}^{r2}$]helicene. The gray and white spheres are carbon and hydrogen atoms, respectively.

We also note the work of the authors of [74] (Figure 28), in which an expanded nano-elicene of the [$1_{zz}^{r2}.2_{zz}^{r2}$]helicene (Figure 28e) as a prototype with almost four coils was made possible.

**Figure 29:**
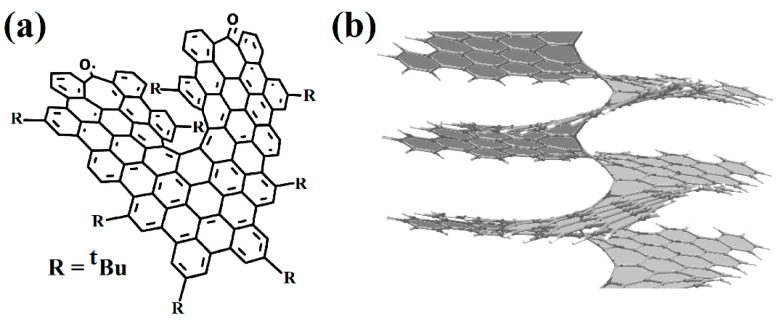
(**a**) One coil of modified, laterally-extended [$1_{zz}^{t}.5_{zz}^{t}$]helicene, synthesized in Ref. [75]. (**b**) Structure of prototypical [$1_{zz}^{t}.5_{zz}^{t}$]helicene. The gray and white spheres are carbon and hydrogen atoms, respectively.

In the mentioned works, there was no goal to obtain nanohelicene with a laterally extended ribbon. This procedure was experimentally carried out in [75] (Figure 29a). A little more than one coil of prototypical [$1_{ac}^{t}.5_{zz}^{t}$]helicene (Figure 29b) was obtained, with the external corner hexagons cut out.

Thus, access to expanded nanohelicenes makes it possible to bypass the steric hindrances of basic [$1_{zz}^{h}.1_{zz}^{h}$]helicene and synthesize oligomers with five coils. The lateral extension of a π-system is also possible, but it is currently limited to only one coil. It can be assumed that the expansion of the shaft for the π-extended ribbon will also make it possible to avoid steric hindrances, and this leads to the construction of promising nanodevices based on expanded laterally extended nanohelicenes.

Nevertheless, despite the fundamental possibility of synthesizing such systems, the methods for their synthesis are still being developed. Therefore, those theoretical and computational works are of particular interest, which helps with determining the possible directions of experimental work for further development.

## 5. Theoretical Consideration of Nanohelicenes with a Fixed Helical Axis Order

### 5.1. Molecular Dynamics

The natural spring topology of nanohelicenes makes the mechanical properties of these systems important. One of the popular methods for studying these properties is the molecular dynamics method, and a large number of works have been devoted to calculating the various properties of nanohelicenes using it. Most of the work used AIREBO inter-atomic potential [76], which demonstrates good accuracy for carbon systems.

**Figure 30:**
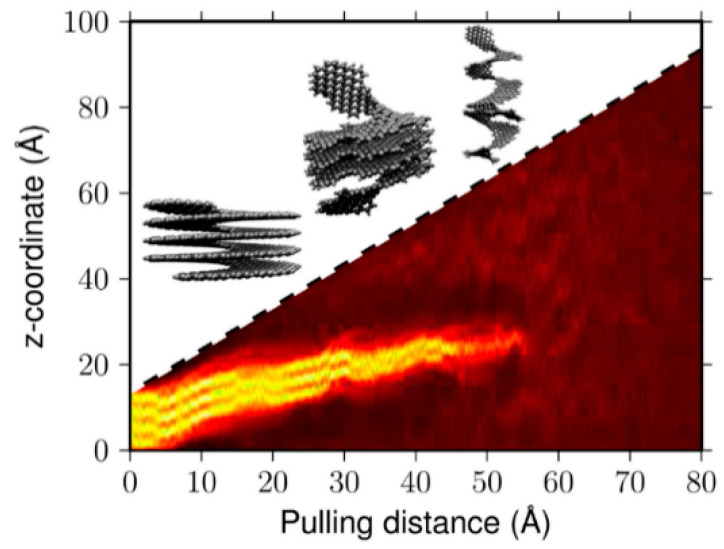
Contour map of the vertical atomic density of [$1_{zz}^{h}.5_{zz}^{h}$]helicene as a function of pulling distance. The density is shown as being Gaussian-broadened with σ = 0.8 Å, with a density scale ranging from zero (black) to 116 Å^−1^ (brightest). The structures shown are snapshots with pulling distances of 0 Å, 20 Å, and 50 Å. Reproduced from Ref. [77].

The behavior of [$1_{zz}^{h}.5_{zz}^{h}$]helicene (four coils) under tension up to 80 Å at 10 and 300 K was studied in [77]. When it is stretched, the layers peel off sequentially, starting with those coils that are fixed from above and below (Figure 30). This behavior differs significantly from a uniform increase in the inter-layer spacing. The retention of interaction between coils with strong local distortions in the π-system is more profitable than a uniform increase in the distance between the coils and maintaining the π-system as flat as possible.

The noted significant inter-layer interaction leads to the so-called non-Hookean behavior of nanohelicenes, which was further investigated in [78], where expanded [$4_{zz}^{h}.4_{zz}^{h}$]helicene (up to 15 coils) was considered. The authors of Ref. [78] show that there are two strain modes. The first one is responsible for the dependence of the energy on the stretching according to Hooke’s law. In this mode, which is realized in the region of small deviations from equilibrium (slightly more than 10%) and when the coils are stretched uniformly, a linear dependence of energy on tension in a logarithmic scale is observed. With a further increase in tension, the second, non-Hookean mode begins to be realized, in which a sequential peeling of the coils is observed. In this mode, the energy increases at a much lower rate with increasing tension.

A nanohelicene of the same morphology (i.e., [$4_{zz}^{h}.4_{zz}^{h}$]) and composed of six coils was considered in [17], and similar, non-Hookean behavior was also observed for it. The most interesting conclusion from [17] is that the investigated [$4_{zz}^{h}.4_{zz}^{h}$]helicene allowed stretching of up to 1800%, which is a unique fact for nano objects. In [19], similar results were obtained when stretching nanohelicene with an (*ac*) termination.

**Figure 31:**
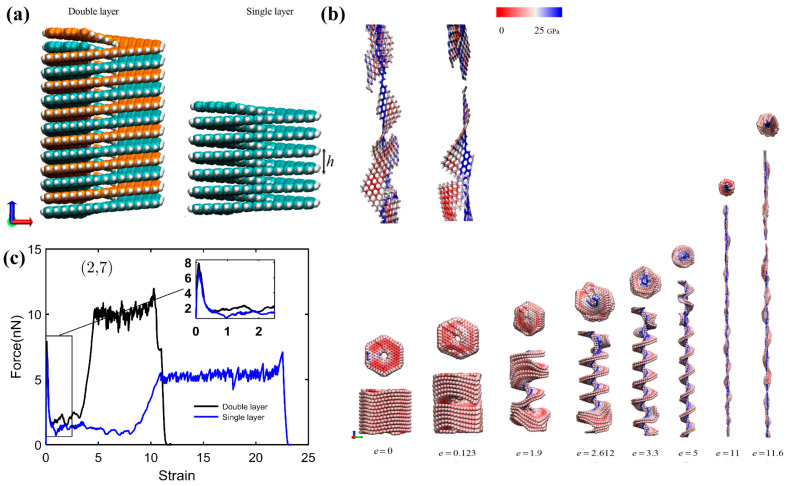
(**a**) Structure of di[$3_{zz}^{h}.3_{zz}^{h}$]helicene and [$3_{zz}^{h}.3_{zz}^{h}$]helicene (six coils); (**b**) Snapshots of deformation of the di[$3_{zz}^{h}.3_{zz}^{h}$]helicene under tensile force. The carbon atoms are colored according to the von Mises stress; (**c**) force diagrams of the di[$3_{zz}^{h}.3_{zz}^{h}$]helicene (“double layer”) and [$3_{zz}^{h}.3_{zz}^{h}$]helicene (“single layer”). Reproduced with permission from Springer Nature, Ref. [79].

The work in [79] is devoted to the MD study of the mechanical properties of [$3_{zz}^{h}.3_{zz}^{h}$]helicene and its double-helix analog, di[$3_{zz}^{h}.3_{zz}^{h}$]helicene (Figure 31a). To the best of our knowledge, this is the first study that addresses the properties of nanohelicenes consisting of a double helix. According to the results of this work, a double helix forms a more rigid nanostructure compared to a single helix of similar morphology (Figure 31b,c). This leads to the rupture of the double-stranded nanohelicene at a much smaller strain (about 1000%) than for the single-stranded version (greater than 2000%). The force required to break the nanohelicene is also about twice as large for a double helix as for a single helix. We also note a feature of the behavior of the double helix in the non-Hookean mode. The neighboring coils of the two helices stick together when stretched due to van der Waals forces, and the resulting structure in this stretching mode is more like a single helix, the coils of which consist of a doubling ribbon (Figure 31b).

In [18], the non-linear static and dynamic behaviors of [$10_{ac}^{h}.14_{ac}^{h}$]helicene were studied, and its implementation for dynamic nanoindentation testing was discussed.

**Figure 32:**
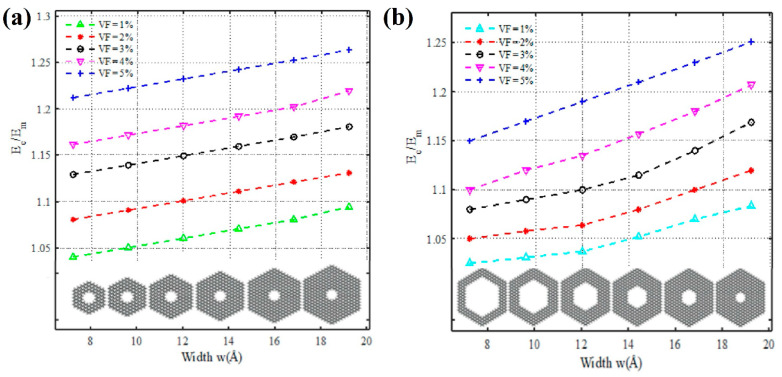
Effects of various sizes of the nanohelicenes on the elastic modulus of the [$m_{zz}^{h}.n_{zz}^{h}$]helicene/polyethylene composite materials for different volume fractions, VF: (**a**) increasing the *n*, (**b**) decreasing the *m*. Reprinted from Ref. [80] (Copyright 2020, with permission from Elsevier).

Further study of mechanical properties has been devoted to the nanocomposites of nanohelicenes with a polymer filler. In particular, [$3_{zz}^{h}.7_{zz}^{h}$]helicene’s nanocomposite (and its variations with different *n* and *m*) with polyethylene was constructed in [80]. The percentage of nanohelicene ranged from 1 to 5%. Thus, the properties of not a 1D object but a 3D polyethylene matrix (in which nanohelicenes with six coils are randomly arranged) were studied. Both the isotropic and anisotropic properties of such composites were studied. It has been shown that an increase in the content of nanohelicene leads to an increase in the relative elastic modulus. An increase in *n* or a decrease in *m* results in the same effect (Figure 32). In [81], the mechanical properties of nanohelicenes with an approximately hexagonal shape and (*ac*) termination were studied as a nanofiller in an epoxy resin matrix. The results also demonstrate a significant increase in the Young modulus of the composite compared to a pure epoxy resin matrix.

**Figure 33:**
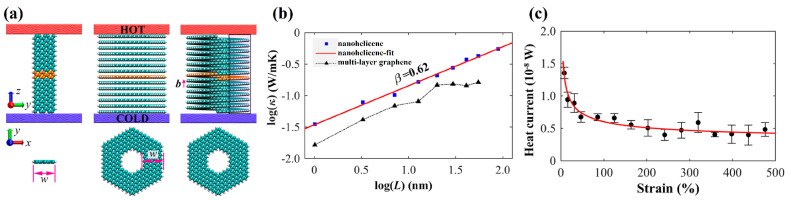
(**a**) Schematic view of the heat transfer scenarios: graphene nanoribbon (**left**), multi-layer graphene (**center**), and nanohelicene (**right**); (**b**) logarithm relationship between thermal conductivity *κ* and sample thickness *L* for nanohelicene and multilayer graphene; (**c**) heat current of nanohelicene as a function of tensile strain. Adapted with permission from Ref. [82] (Copyright 2018 American Chemical Society).

In the next group of works under our consideration, the thermal characteristics of nanohelicenes were studied by MD method. In [82], the thermal conductivity of [$4_{zz}^{h}.4_{zz}^{h}$]helicene was studied by non-equilibrium molecular dynamics (NEMDs) in comparison with a stack of corresponding nanorings (simulating a graphite nanorod with terminal carbons saturated by hydrogens) and with a graphene nanoribbon of the same thickness (Figure 33a). It has been shown that nanohelicene has a higher conductivity than a stack of nanorings (Figure 33b). However, more significant is the fact that nanohelicene exhibits a power-law dependence of conductivity on length (i.e., the number of coils), which is similar to the thermal properties of graphene nanoribbon. In other words, nanohelicenes are expected to exhibit super-diffusive heat transport and divergent thermal conductivity, similar to graphene nanoribbon. The spring topology of nanohelicene makes it possible to change the thermal conductivity with a simple stretch compression. In particular, a threefold decrease in heat current was obtained at sufficiently large tensions (greater than 100%). Compression, as expected, leads to an improvement in the thermal conductivity of nanohelicene (Figure 33c).

Further research in this direction was carried out in [83] for [$3_{zz}^{h}.3_{zz}^{h}$]helicene, along with calculations of other systems with helical topology. It has been shown that such nanohelicene can be applied as a perspective thermal nanodiod. In addition, the effect of hydrogenation on thermal rectification has been discussed. The latter, along with nitrogen doping, was further also studied in work [84] for H-unsaturated nanohelicenes with [$3_{zz}^{h}.5_{zz}^{h}$], [$3_{zz}^{h}.3_{zz}^{h}$], and [$2_{zz}^{h}.3_{zz}^{h}$] morphologies along with the studies of thermal conductance.

### 5.2. DFTB and DFT

The results obtained by the MD method are devoted to the study of the features of the atomic structure of nano-helicenes associated with the spring topology. However, the features due to the simultaneous presence of a continuous π-system and a helical spring topology require studies of the structure of nanohelicenes, at least, by the semi-empirical DFTB method, or even ab initio DFT.

As already mentioned, the first work in which the electronic structure of infinite [$1_{zz}^{h}.1_{zz}^{h}$]helicene and [$1_{ac}^{h}.2_{ac}^{h}$]helicene was considered at the DFTB level was [39]. The electronic band pictures show that these structures have semiconductor and metallic properties, respectively.

**Figure 34:**
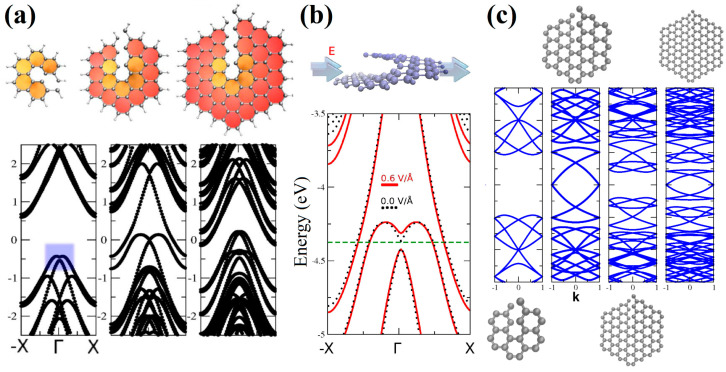
(**a**) Top view of the structure and the electronic band structures of [$1_{zz}^{h}.n_{zz}^{h}$]helicenes with *n* = 1, 2, and 3; (**b**) (red lines) band structure for [$1_{zz}^{h}.2_{zz}^{h}$]helicenes under the influence of an external electric field *E* = 0.6 V/Å applied perpendicularly to the axial direction. The horizontal line marks the Fermi energy (not shifted to zero). The dotted black lines are the energy dispersion for the null electric field. (**c**) Top view of the structure and the electronic band structures of [$1_{zz}^{h}.n_{zz}^{h}$]helicenes with *n* = 1, 2, 3, and 4 obtained using the simple tight-binding model. Energies are shown in units of hopping parameter *t*, and Fermi energy is set at zero. Adapted from Ref. [16].

Consideration of nanohelicenes from the standpoint of topological properties due to helicity was started in [16] by using DFT with pure GGA PBE functional calculations. [$1_{zz}^{h}.1_{zz}^{h}$]helicene, [$1_{zz}^{h}.2_{zz}^{h}$]helicene, and [$1_{zz}^{h}.3_{zz}^{h}$]helicene were considered, of which the latter two had semi-metallic properties (Figure 34a). The most interesting result of [16] was the manifestation of the intrinsic Rashba effect due to the absence of inversion symmetry. The Rashba effect arises from spin-orbit interaction. Despite the smallness of this interaction in carbon systems, it is important for applications due to the possibility of controlling an external electric field (Figure 34b). Additionally, nanohelicenes of morphology [$1_{zz}^{h}.n_{zz}^{h}$] with *n* = 1, 2, 3, and 4 were studied by using the DFTB method in [16] (Figure 34c).

Based on the obtained result on the metallicity of [$1_{zz}^{h}.2_{zz}^{h}$]helicene and [$1_{zz}^{h}.3_{zz}^{h}$]helicene, the possibility of these nanohelicenes working as nanosolenoids, i.e., create a magnetic field when an electric current passes through them, was found. The obtained values of magnetic field intensity, *B*, can reach very large values: *B* ~ 10^3^ T.

**Figure 35:**
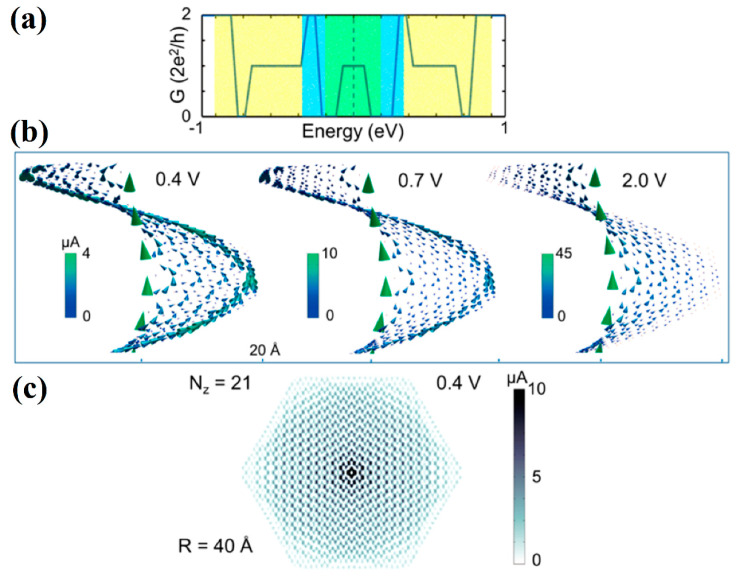
[$1_{zz}^{h}.8_{zz}^{h}$]helicene: (**a**) transmission coefficient as a function of energy; (**b**) spatial current distribution integrated over the various energy ranges of the previous panel—left for green region (bias *V* = 0.4 V), middle for green plus blue region (*V* = 0.7 V), right for green plus blue plus yellow region (*V* = 2 V); (**c**) bright-dark map of current distribution for [$1_{zz}^{h}.20_{zz}^{h}$]helicene, and a bias of 0.4 V. Adapted with permission from Ref. [56] (Copyright 2015 American Chemical Society).

Further studies on nanohelicenes as nanosolenoids were presented in [56] (Figure 35). The magnetic field intensity values obtained in this work were smaller, *B* ~ 1 *T*, but this is also a very large value (Figure 35). In this work, the hexagonal nanohelicenes of two terminations were studied by DFTB, and, for the first time, it was shown that there are selection rules for nanohelicenes that indicate which structures will be metals and which will not. It has been shown that the presence of metallic properties is due only to the width of the ribbon. The values of the electronic band gap qualitatively depend on the shaft radius.

**Figure 36:**
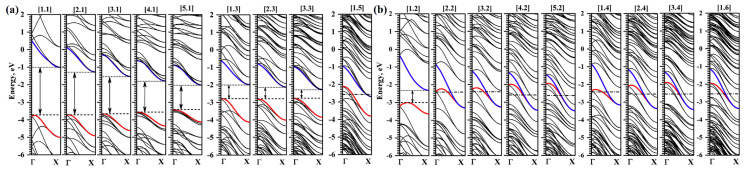
(**a**) Electronic band structure of [$m_{zz}^{h}.1_{zz}^{h}$], [$m_{zz}^{h}.3_{zz}^{h}$] and [$m_{zz}^{h}.5_{zz}^{h}$]helicenes with *R* < 7 (see Equation (5)); (**b**) electronic band structure of [$m_{zz}^{h}.2_{zz}^{h}$], [$m_{zz}^{h}.4_{zz}^{h}$], and [$m_{zz}^{h}.6_{zz}^{h}$]helicenes with *R* < 8. In the case of the [$1_{zz}^{h}.2_{zz}^{h}$]helicene, the α-spin electron band structure is shown. The upper valence band and lower conduction band branches are in red and blue, respectively. The dash and dot lines show the top of the valence band and the bottom of the conduction band, respectively. The arrows denote indirect band gaps. The dash-dotted line indicates Fermi energy. Reprinted from Ref. [12] (Copyright 2019, with permission from Elsevier).

For zigzag-terminated hexagonal nanohelicenes, the selection rules were confirmed by DFT calculations using a hybrid functional PBE0 [12]. In this work, expanded lateral-extended [$m_{zz}^{h}.n_{zz}^{h}$]helicenes were considered with all possible combinations of *m* and *n* indices satisfying the condition *m* + *n* < 8. It was shown that the fundamental possibility of the appearance of metallic properties is due to group theoretical relations at the boundary of the Brillouin zone, and this is determined by the evenness of the index *n* (i.e., the width of the ribbon in the case of nanohelicenes with (*h zz*) morphology). If *n* is odd, then nanohelicene is a semiconductor (Figure 36a). If *n* is even, then nanohelicene is a metal (Figure 36b). The index *m* affects only the quantitative value of the band gap in the case of an odd *n*. An increase in *m* for a given *n* reduces the band gap. Interestingly, in this case, one can expect that, at a sufficiently large *m*, nanohelicenes will exhibit metallic properties even with an odd *n*, although, topologically, the band structure will correspond to a semiconductor (see, for example, the band structure of [$1_{zz}^{h}.5_{zz}^{h}$]helicene on Figure 36a).

**Figure 37:**
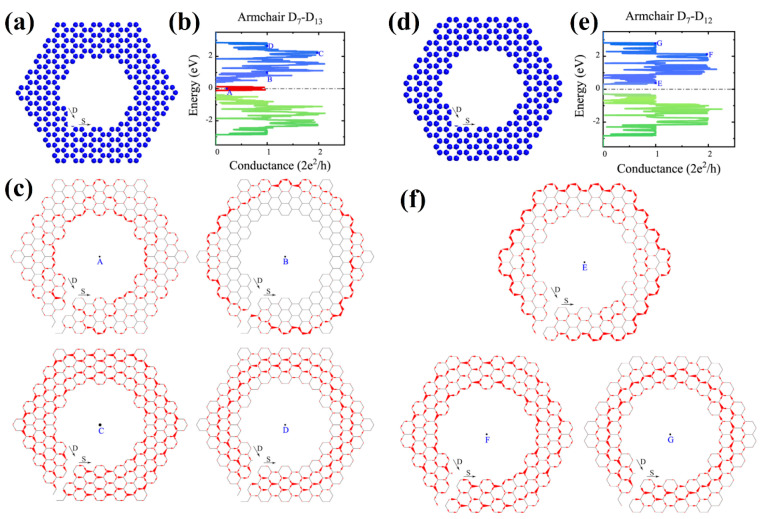
(**a**) Top view of the atomic structure, (**b**) conductance, and (**c**) microscopic current distributions of modified [$5_{ac}^{h}.4_{ac}^{h}$]helicene. The current distributions are plotted for four different conductance states A, B, C, and D, for which the energy value is labeled in the (**b**) panel. (**d**) Top view of the atomic structure, (**e**) conductance, and (**f**) microscopic current distributions of modified [$5_{ac}^{h}.3_{ac}^{h}$]helicene. The current distributions are plotted for three different conductance states E, F, and G, for which the energy value is labeled in the (**e**) panel. Reprinted from Ref. [85] (Copyright 2022, with permission from Elsevier).

In [85], the selection rules obtained for nanohelicenes [12,56] were also confirmed by DFTB calculations for both terminations. Conductance and current distribution for one coil in nanohelicenes with [$5_{ac}^{h}.4_{ac}^{h}$] and [$5_{ac}^{h}.3_{ac}^{h}$] morphology with a modified inner edge was also considered. Some of the microscopic current distributions were presented, depending on their energy (Figure 37). It should be noted that in [85], the coils of nanohelicenes were located at a considerable distance from each other, and only the current inside the ribbon was considered; the inter-layer current was not taken into account, although in [44,45,46], it was noted that the current passed predominantly through inter-layer ways.

**Figure 38:**
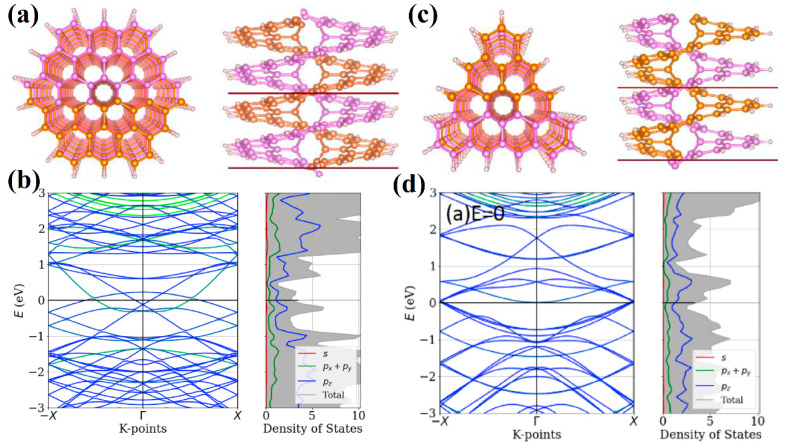
(**a**) The top (**left**) and side (**right**) views, (**b**) band structure (**left**), and density of states (**right**) of di[$1_{zz}^{h}.2_{zz}^{h}$]helicene; (**c**) top (**left**) and side (**right**) views, (**d**) band structure (**left**), and density of states (**right**) of di[$1_{zz}^{t6}.2_{zz}^{t6}$]helicene. Reprinted from Ref. [86] (Copyright 2021, with permission from Elsevier).

In [86], the DFT using a GGA PBE functional and Grimme semi-empirical dispersion correction method was used to study the stability and electronic properties of hexagonal [$1_{zz}^{h}.2_{zz}^{h}$]helicene and trigonal [$1_{zz}^{t6}.2_{zz}^{t6}$]helicene and their analogs with two helices, i.e., di[$1_{zz}^{h}.2_{zz}^{h}$]helicene and di[$1_{zz}^{t6}.2_{zz}^{t6}$]helicene (Figure 38a,c). According to the calculations, all structures had metallic conductivity (Figure 38b,d); however, the band gap opens up when an electric field is applied. In the case of di[$1_{zz}^{h}.2_{zz}^{h}$]helicene, the fine splitting of bands due to SOC has been observed between the Brillouin zone center and zone boundary, which is in contrast to di[$1_{zz}^{t6}.2_{zz}^{t6}$]helicene.

**Figure 39:**
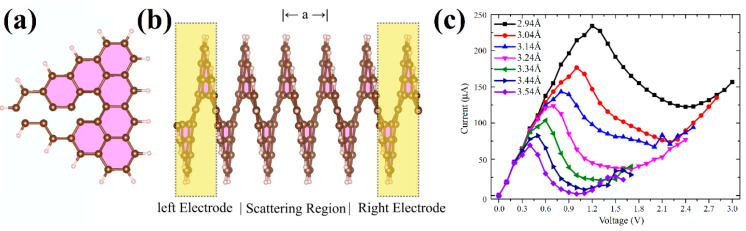
(**a**) Top view of the atomic structure of [$1_{zz}^{t6}.2_{zz}^{t6}$]helicene; (**b**) two-probe system based on [$1_{zz}^{t6}.2_{zz}^{t6}$]helicene; (**c**) *I/V* curves for different distances between coils. The 3.24 Å is an equilibrium value. Used with permission of IOP Publishing, Ltd., from Ref. [87]; permission conveyed through Copyright Clearance Center, Inc.

Electron transport through a single [$1_{zz}^{t6}.2_{zz}^{t6}$]helicene (four coils) was also theoretically studied in [87] using the DFT with functional GGA PBE (Figure 39a,b). Calculations have shown that nanohelicenes of this morphology exhibit negative differential resistance in a certain region on the *I*/*V* curve, and this region strongly depends on tensile strength, which makes this property promising for electro-mechanical devices (Figure 39c).

It should be noted that the mentioned studies on electron transport did not take into account the possible manifestation of magnetic properties by nanohelicenes. This possibility must be taken into account, at least on the basis of the results of the theoretical consideration of graphene nanoflakes, which are the basic “building blocks” for the construction of nanohelicenes.

**Figure 40:**
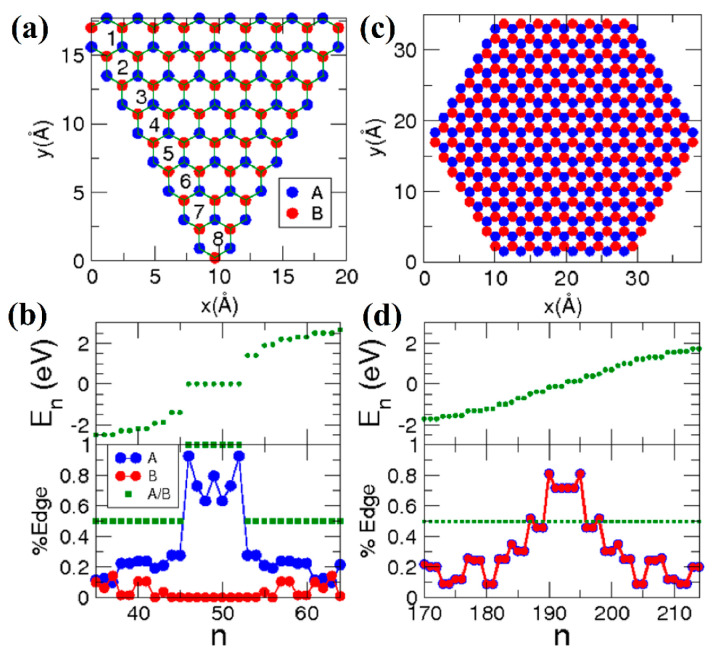
Atomic structure of zigzag-terminated (**a**) trigonal and (**c**) hexagonal graphene nanoflakes; sublattice resolved edge content and sublattice polarization of (**b**) trigonal and (**d**) hexagonal nanoflakes. The atoms of the *A* and *B* sublattices are blue and red, respectively. With permission from Ref. [57] (Copyright 2007 American Physical Society).

The properties of graphene nanoflakes with trigonal and hexagonal zigzag termination at the DFTB level were considered in [57,58]. Magnetism in these nanoflakes is determined by the already-mentioned Lieb theorem [55]. In particular, trigonal zigzag-terminated graphene nanoflakes have a different number of atoms in the *A* and *B* sublattices, *N_A_* ≠ *N_B_*, and therefore exhibit ferromagnetic properties (Figure 40a,b). In the case of hexagonal zigzag-terminated nanoflakes, the number of atoms in each sublattice is the same, *N_A_* = *N_B_*, and they are arranged symmetrically. Therefore, such systems can be either paramagnetic (diamagnetic) or antiferromagnetic, which is determined by the parameter *U* of the Hubbard model. In [57], a comparison of the results of the DFT calculations reveals that *U* = 1.5 eV. In this case, hexagonal graphene nanoflakes with an edge width of less than nine hexagons are paramagnetic, while larger ones exhibit antiferromagnetic ordering (Figure 40c,d).

**Figure 41:**
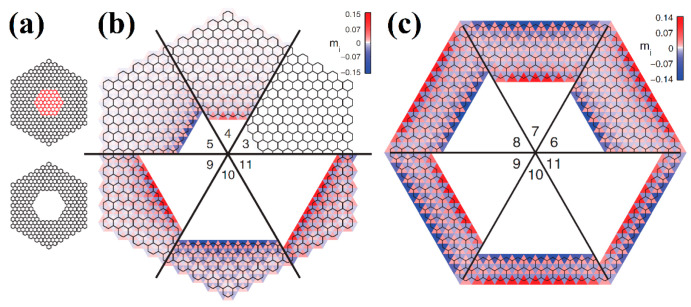
(**a**) Formation of the graphene nanoring of [$4_{zz}^{h}.9_{ac}^{h}$] morphology (bottom) from hexagonal *ac*-terminated graphene nanoflake with *R* = 13 (top). Distribution of local magnetic moments *m_i_* of graphene nanorings of (**b**) [$m_{zz}^{h}.n_{ac}^{h}$] morphologies with *R* = 17, and (**c**) [$m_{zz}^{h}.n_{zz}^{h}$] morphologies with *R* = 13. To obtain the *m* index, add one to the value in the sextant of the ring. The majority spin is labeled by both the orientation and color of a triangle centered at an atomic site *i*. The values of *m_i_* are proportional to the color intensity. With permission from Ref. [59] (Copyright 2013 American Physical Society).

Armchair-terminated trigonal graphene nanoflakes were mentioned in [58] but have not been studied in detail. In [59], graphene hexagonal nanoflakes with armchair edge termination were studied within the framework of the Hubbard model. They exhibit diamagnetic properties regardless of the length of the edge. Further, in the same work, hexagonal nanorings of both terminations with a hexagonal inner shaft of a zigzag termination region were studied (Figure 41a). It was shown that cutting out a sufficiently large shaft leads to the appearance of antiferromagnetism, even if the outer edge has an armchair termination (Figure 41b,c).

Similar behavior of graphene nanoflakes with different terminations is expected if we compare it to the properties of graphene nanoribbons of the corresponding terminations. The armchair-terminated nanoribbons do not exhibit magnetic properties, while zigzag-terminated nanoribbons exhibit antiferromagnetic ordering. Spin densities of opposite signs are localized at opposite edges of the nanoribbon [88]. The saturation of nanoribbon edges with hydrogen does not change the magnetic properties; the zigzag-terminated nanoribbon remains antiferromagnetic [89]. It is also important to note the fact that it is known in organic chemistry, whereby acene molecules, which can be considered as hydrogen-saturated graphene zigzag-terminated nanoribbons of finite length, are unstable with increasing length [90].

**Figure 42:**
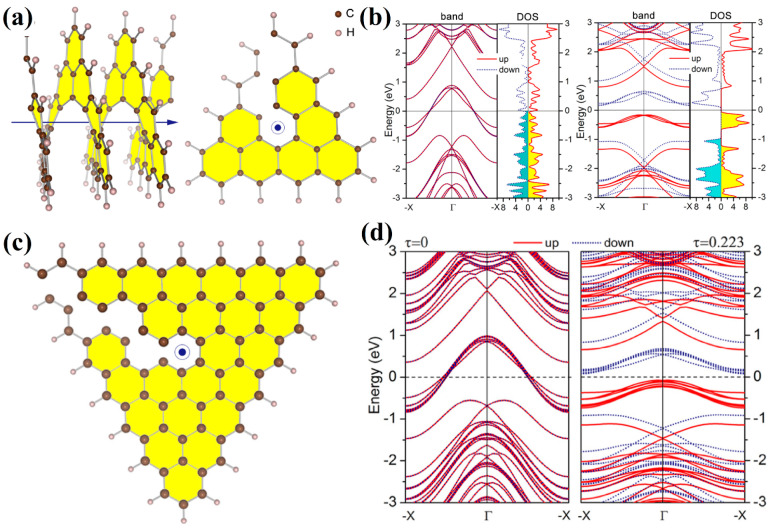
[$1_{zz}^{t6}.2_{zz}^{t6}$]helicene: (**a**) side view (**left**) and top view (**right**); (**b**) spin-resolved band structure and electron density of states at equilibrium (**left**) and under the tensile strain τ = 0.235 (**right**). [$1_{zz}^{t6}.3_{zz}^{t6}$]helicene: (**c**) top view; (**d**) spin-resolved band structure at equilibrium (**left**) and under the tensile strain τ = 0.223 (**right**). Adapted from Ref. [91].

Due to the conclusions obtained in the analysis of graphene nanoflakes, it is easy to understand the results of the theoretical considerations of nanohelicenes based on them. Indeed, [$1_{zz}^{t6}.2_{zz}^{t6}$]helicene and [$1_{zz}^{t6}.3_{zz}^{t6}$]helicene (Figure 42a,c), which were studied in [91] at the level of DFT with functional GGA PBE, are metallic in equilibrium geometry and spin polarization is absent. However, a small axial tension leads to a phase transition into a ferromagnetic semiconductor of both nanohelicenes (Figure 42b,d). This corresponds to the previously mentioned qualitative result obtained on the basis of graphene nanoflakes and due to the fact that the number of atoms of sublattices *A* and *B* is not equal to each other. 

The effect of the peripheral modification of [$1_{zz}^{h}.1_{zz}^{h}$]helicene edges on the magnetic properties of nanohelicenes were studied in detail in [92] at the level of DFT with GGA PBE or hybrid functional HSE. The influence of axial strains on the possibility of phase transitions to the magnetic state and their influence on the optical properties from the point of view of the direct/indirect band gap was considered. Note that, in addition to asymmetric peripheral modifications, modifications that lead to [$1_{ac}^{h}.2_{ac}^{h}$]helicene, [$1_{zz}^{t6}.2_{zz}^{t6}$]helicene, and [$1_{zz}^{r6}.1_{zz}^{r6}$]helicene were also studied. These structures are minimal representatives of the nanohelicenes of the corresponding morphology. The results on trigonal [$1_{zz}^{t6}.2_{zz}^{t6}$]helicene agree with the results of [91], manifesting ferromagnetic properties under tension. The absence of magnetic properties in the case of [$1_{ac}^{h}.2_{ac}^{h}$]helicene is consistent with the results of [59] on armchair-terminated nanoflakes and graphene nanoribbons with the same termination [88]. We also note the absence of magnetic properties for rhombic [$1_{zz}^{r6}.1_{zz}^{r6}$]helicene, which was not previously considered in theoretical studies.

**Figure 43:**
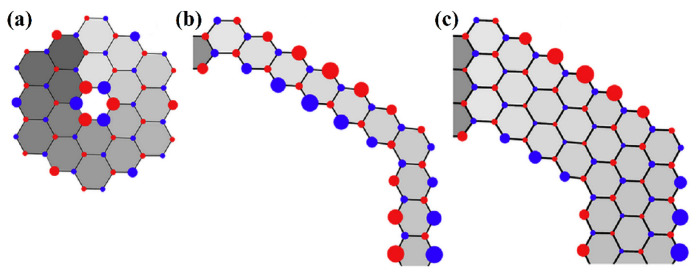
The Mulliken spin density of (**a**) [$1_{zz}^{h}.2_{zz}^{h}$]helicene, (**b**) [$6_{zz}^{h}.1_{zz}^{h}$]helicene fragment, and (**c**) [$4_{zz}^{h}.3_{zz}^{h}$]helicene fragment. The diameter of the red and blue spheres is proportional to the positive and negative values, respectively. H atoms are omitted for clarity. Reprinted from Ref. [12] (Copyright 2019, with permission from Elsevier).

The magnetic properties of hexagonal zigzag-terminated [$m_{zz}^{h}.n_{zz}^{h}$]helicenes were studied in [12]. The results obtained show a two-fold way of manifesting antiferromagnetism (Figure 43), which have different theoretical justifications.

First, antiferromagnetic ordering manifested itself in a complete analogy with the results of the graphene nanoflakes and nanorings [59] of the corresponding morphology at large nanohelicene edge length, *R* (see Equation (5)). Namely, at *R* > 6 hexagon rings, nanohelicene becomes antiferromagnetic, with the spin polarization of the edges being similar to that of graphene nanoribbons of a zigzag termination [88] and acenes [90]. The manifestation of this polarization does not depend on *n* and *m* but depends only on their sum, *R* (Figure 43b,c). This qualitatively agrees with the antiferromagnetism obtained for sufficiently large nanorings, in which *R* > 9 [57].

The second mechanism for the emergence of antiferromagnetic ordering in nanohelicenes is due to the effects associated with the nature of nanohelicenes as 1D nanostructures. As it is known, the metallic state in the case of 1D periodic structures is unstable if electronic bands at the edge of the Brillouin zone are coupled and can undergo spontaneous symmetry breaking. This results in the uncoupling of these bands and a transition to a semiconductor state (i.e., metal-to-insulator transition, MIT). The Peierls MIT [93] is due to the electron-phonon interaction and results in shifts of the initially symmetrically equivalent neighboring atoms, with a doubling of the elementary cell. The Mott-Hubbard MIT [94] occurs due to electron-electron correlation, and symmetry reduction occurs due to the spin degrees of freedom. In other words, the initially symmetrically equivalent neighboring atoms have a different spin projection in the Mott-Hubbard MIT case.

As already mentioned, [$m_{zz}^{h}.n_{zz}^{h}$]helicenes with an even *n* have metallic properties, and, according to Figure 36b, the coupling of the electronic bands at the X point of the Brillouin zone is present. Therefore, such nanohelicenes are unstable with respect to spontaneous symmetry breaking. According to DFT calculations with hybrid functional PBE0, the minimal nanohelicene with an even *n*, [$1_{zz}^{h}.2_{zz}^{h}$]helicene, becomes an antiferromagnetic semiconductor due to spontaneous symmetry breaking according to the Mott-Hubbard MIT mechanism [12] (Figure 43a). Larger nanohelicenes of this type remain metallic in the equilibrium state, but small axial stretching leads to a phase transition to the antiferromagnetic semiconductor state.

As already mentioned, a phase transition of this kind to a semiconductor state with ferromagnetic ordering also experiences trigonal [$1_{zz}^{t6}.2_{zz}^{t6}$]helicene [86]. This can also be considered a consequence of spontaneous symmetry breaking by the Mott-Hubbard MIT mechanism.

The MIT in expanded [$m_{zz}^{h}.1_{zz}^{h}$]helicenes with H-unsaturated terminal carbon atoms (with *m* = 2, 3, 4, 5) was studied in [95] by DFT using functional GGA PBE. All considered systems were found to be metallic, possibly due to unsaturated σ-bonds, but undergoing MIT at sufficient axial straightening, which is similar to the results for nanohelicenes with H-saturation. In further work [96], which concerns H-unsaturated nanohelicene of [$2_{zz}^{h}.1_{zz}^{h}$] morphology, it was shown that a spin-polarized current along with an electrical one. Moreover, at some voltages, the spin polarization reached 100%, showing the great application potential of this system.

Thus, quantum chemical calculations of nanohelicenes demonstrate the unique electronic and magnetic properties of these nanostructures due to the combination of helical topology and a continuous π-system. Helical topology implies a non-centrosymmetric structure and the manifestation of Rashba spin splitting. A more detailed analysis of the spatial and magnetic symmetry conditions makes it possible to divide the 1651 magnetic space groups into seven different spin-splitting prototypes (SST) [97]. The analysis of SSTs for all 394 magnetic rod groups was carried out in [98]. An important result of this analysis is the fundamental possibility of spin splitting in the absence of spin-orbit interactions; however, such studies have not yet been carried out for nanohelicenes.

We also note that, due to helical topology, in almost all the theoretical works considered, a strong dependence of the electronic, magnetic, and optical properties on the application of axial stress was observed. However, in all these works, one more degree of freedom, namely torsion deformation, was not considered at all. As will be clear from the discussion that follows, torsion stresses have the same or even greater influence on the tuning of nanohelicene properties when compared to axial stresses. Moreover, taking this degree of freedom into account enables researchers to find the true minimum energy of nanohelicenes as nanostructures with helical periodicity.

## 6. Theoretical Consideration of Nanohelicenes in the Paradigm of Helical Periodicity

### 6.1. Line Group Symmetry

**Figure 44:**
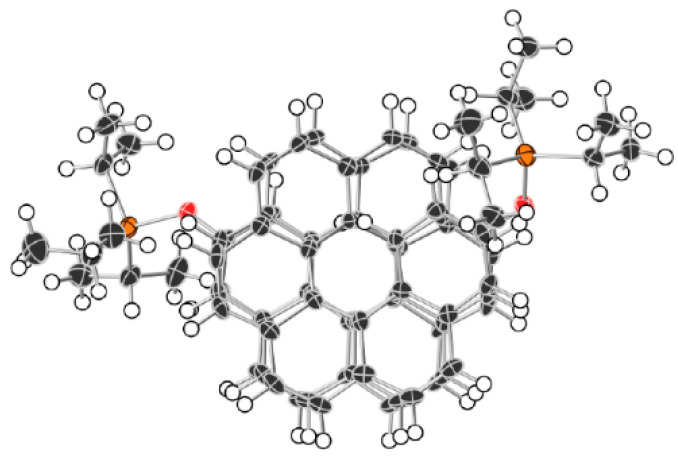
Top view of 3,34-bis(triisopropylsilyloxy)[16]helicene from X-ray crystallographic data. With permission from Ref. [23] (Copyright 2015 John Wiley and Sons).

When constructing nanohelicenes based on graphene nanorings and nanoflakes, the symmetry of the resulting structures was described by the corresponding rod groups. This leads to the fact that all the coils of nanohelicene are translationally equivalent to each other. In other words, the translational period is equal to the height of the coil. However, such an assumption is a simplification of the real picture; from the crystallographic data of molecular [16]helicene (Figure 44), it is already known that the coils are twisted relative to each other by a certain angle [23].

Therefore, the description of the symmetry of nano-helicenes requires the use of the apparatus of line symmetry groups. These groups describe the symmetry of systems that are periodic in one direction without any restriction on the helical axis order [99]. These groups are also named spiral groups in [100,101]. Further, the factorization of the line groups as a weak direct product of cyclic groups proved to be an essential property of both commensurate and incommensurate line groups [102,103].

Here, we will consider the basic concepts of the theory of line symmetry groups, which are necessary for understanding any further discussion. A detailed description of the construction of the line symmetry groups and their representations, along with some practical results, is presented in the monography [104].

The theory of line symmetry groups is based on helical axis operations, *Z* = (*C_Q_*|*f*), called *generalized translations*, instead of pure translations, *T*(*A*) with period *A*. *Q* specifies the order of the helical axis, and *f* is the *partial translation* along the helical axis. The *Z* operation acts on the *monomer*, mapping it onto the adjacent ones and, thus, generating an infinite cyclic group, ***Z***. Operation *C_Q_* is the rotation around the helical axis according to *rotation angle*:

(6) $$\varphi =\frac{2\pi }{Q}=\frac{360{}^{\circ}}{Q}$$

(in radians or degrees, respectively).

Thus, the 6_1_ operation, *Z* = (*C*_6_|*f*), implies *Q* = 6 and rotation angle is

(7) $$\varphi \left(6_{1}\right)=\frac{360{}^{\circ}}{Q}=60{}^{\circ}$$

Partial translation is

(8) $$f\left(6_{1}\right)=\frac{A}{6}$$

Applying this operation six times will result in a 360° rotation and translation in period *A*. This is a case of a simplified description of nanohelicenes. Based on the experimental fact [23] that the second coil of [16]helicene is twisted relative to the first one, it is easy to see that, in a real situation, the monomer turns through a rotation angle of φ>60°. Then, one can generalize the concept of helical axis order using Equation (6), i.e.,

(9) $$Q=\frac{360{}^{\circ}}{\varphi }>1$$

Obviously, in this case, it is necessary to get away from understanding the order of the helical axis as an integer. Moreover, since no restrictions are imposed on φ that make it an integer or at least a rational number, we can then consider φ to be real (i.e., possibly irrational), which means that the order of the helical axis will be the same.

Thus, we must assume that the true symmetry of nanohelicenes is determined by some (most likely irrational) *Q* and some translation *f*, which determine the operation *Z* from the helical group ***Z***. If a 1D nano object is invariant with respect to the operations of ***Z***, then such an object is *helically periodic*.

Group ***Z*** is an invariant subgroup of the line symmetry group, ***L***. The group ***L*** also includes the monomer point symmetry group, ***P***, which is consistent with the operations of ***Z***. The group ***L*** factorizes into a semi-direct product of ***Z*** and ***P***:

(10) L=Z∧P

A total of 13 families of non-magnetic and 81 families of magnetic line groups are known (for more details, see [104]). Nanohelicenes (and other systems with an irrational order of the helical axis) are described by those families in which there is no center of inversion or symmetry planes, namely the first and fifth families.

The first family includes line symmetry groups with ***P*** = *C_n_*, where *n* is the order of the rotation axis co-directed with the helical axis. If *n* = 1, then nanohelicene is single helix, i.e., ***L*** = ***Z***; if *n* >1, then nanohelicene consists of *n* helices. The fifth family consists of line symmetry groups with ***P*** = *D_n_*, which additionally include a second-order axis, *U*, perpendicular to the helical axis. For single-helix nanohelicene, ***P*** = *D*_1_.

In order for the line symmetry group to have translation operations, it is necessary that an integer *q* exists such that

(11) $${\left(C_{Q}|f\right)}^{q}=\left(C_{Q}^{q}|qf\right)=(E|A)$$

This condition is satisfied if *Q* is a rational number:

(12) $$Q=\frac{q}{r}$$

Here, *q* and *r* are coprime integers, and *q* ≥ *r*.

Any Cauchy sequence of rational numbers converges to a real number (in particular, an irrational one). Therefore, to study 1D nanostructures with an irrational *Q*, it is possible to use quantum chemical programs based on the translational periodicity that only exists for a rational *Q*.

The paper [105] presents an algorithm that allows for the quantum-chemical calculation of the properties of helically periodic nanostructures with an irrational *Q*. It is based on the interpolation of the properties obtained for translationally periodic structures with a rational *Q*. This algorithm works best when using the CRYSTAL program [106,107]. The CRYSTAL code uses a Gaussian-type atomic basis set that is most beneficial for sub-periodic 1D systems. Indeed, the maximal use of symmetry (including a rational *Q*) that is implemented in all steps of the calculations leads to drastically reduced computation time and memory.

In the case of an irrational *Q*, the classification of the irreducible representations of the line symmetry group by the translational subgroup ***T***(*A*) is impossible since the translational Brillouin zone collapses into a point. Therefore, for helically periodic systems, a classification based on partial translation, *f*, is used:

(13) $$A_{\tilde{k}}\left(Z\right)=e^{i\tilde{k}f},\;\tilde{k}=\left(-\frac{\pi }{f},\frac{\pi }{f}\right].$$

Interval $\left(-\frac{\pi }{f},\frac{\pi }{f}\right]$ is called the helical Brillouin zone, and $\tilde{k}$ is a helical wave vector. 

Obtaining the electronic band picture in the helical Brillouin zone based on the results of calculations using the CRYSTAL program is presented in [108].

The algorithm presented in [105] is general, and besides nanohelicenes, it was used for the quantum chemical study of other 1D nanostructures, such as polytwistane [109], GaS nanotubes [110], and helical polyacetylenes [111].

If the considered nanostructure manifests magnetic ordering, the magnetic line groups should be used [102]. In the case of the first family of line groups and ***P*** = C_1_, only *klassengleiche* magnetic line groups are possible:

(14) $$L_{mag}=Z{}^{\prime }+\theta Z{}^{\prime }.$$

Here, *θ* is the time reversal operation, and ***Z’*** is a subgroup of ***Z*** of index 2. Group ***Z = L_mag_*** is a line symmetry group of an initial state of a nanostructure.

If *Q* and *f* are the parameters of the initial state, then the dimerization of the neighboring monomers leads to a doubling of *f* and a halving of the helical axis order of *Q*:

(15) $$Q^{\prime }=\frac{Q}{2},\;f^{\prime }=2f.$$

The *Q’* and *f’* are parameters of ***Z’*** helical group.

### 6.2. Helical Periodicity of Nanohelicenes: DFT Studies

**Figure 45:**
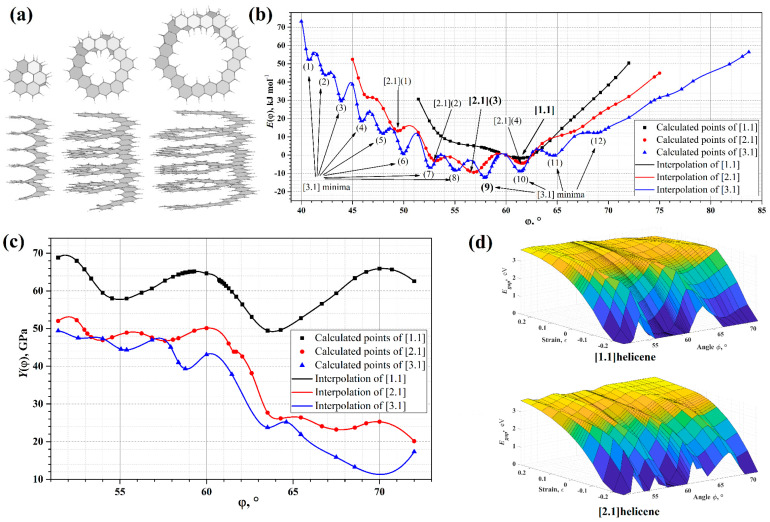
(**a**) Top and side views of the atomic structure, (**b**) the energy *E*(φ) diagrams, and (**c**) Young’s moduli *Y*(φ) dependences of [$m_{zz}^{h}.1_{zz}^{h}$]helicenes with *m* = 1, 2, 3; (**d**) the two-parametric electronic band gap *E*_gap_(φ, ε) dependences of [$1_{zz}^{h}.1_{zz}^{h}$]helicene (top) and [$2_{zz}^{h}.1_{zz}^{h}$]helicene. Reprinted from Ref. [105] (Copyright 2022, with permission from Elsevier).

Quantum chemical calculations of nanohelicenes that take helical periodicity into account were first performed in [105]. The calculations were based on an approach that uses a certain range of rational *Q* values around *Q* = 6 to obtain the torsion energy curve of nanohelicene. A number of [$m_{zz}^{h}.1_{zz}^{h}$]helicenes with *m* = 1, 2, and 3 were considered at the DFT level with the hybrid PBE0 functional (Figure 45a). The electronic properties with geometry optimization were calculated in intervals of orders *Q* of the helical axis, including the crystallographic *Q* = 6. According to previous calculations [12], these nanohelicenes have a simple electronic structure and are diamagnetic semiconductors.

As already mentioned, one cannot expect that after the construction of nanohelicenes, according to the rules of Section 3, the symmetry will remain crystallographic. The order of the helical axis can be irrational, and the systems can have only helical periodicity but not a translational one. Nevertheless, general considerations based on, among other things, the rigidity of the carbon π-system of nanohelicenes allow one to expect that the order of the helical axis will be approximately equal to 6. This has been tacitly assumed in all previous theoretical works.

The calculation results [105] demonstrate that the curve of energy dependence on torsion deformation of [$1_{zz}^{h}.1_{zz}^{h}$]helicene has only one minimum corresponding to *Q* ≈ 5.86, i.e., rotation angle φ ≈ 61.43° (Figure 45b). The obtained rotation angle value corresponds to the structure in which the coils are *overtwisted* (*Q* < 6), which is in good agreement with the experimental data on molecular [16]helicene (Figure 44). The small difference between the true order of the helical axis and *Q* = 6 allows us to see that taking into account helical periodicity only refines the qualitatively correct picture obtained at *Q* = 6.

Unlike [$1_{zz}^{h}.1_{zz}^{h}$]helicene, nanohelicenes with *m* = 2 and 3 in their global energy minimum have a structure in which the coils are *undertwisted*, i.e., *Q* > 6 (Figure 45b). For [$2_{zz}^{h}.1_{zz}^{h}$]helicene, Q ≈ 6.33, and for [$3_{zz}^{h}.1_{zz}^{h}$]helicene, Q ≈ 6.22. The values of *Q* do not show any explicit dependence on *m* and, obviously, are the result of the influence of several factors at once: (i) the tendency of a distorted π-electron system to be flat; (ii) the tendency of the hexagons formed by sp^2^-hybridized carbon atoms to be regular; (iii) the tendency of neighbor coils to form an AB arrangement similar to a graphite structure.

Simultaneous modeling of torsion and axial deformations is also possible, which opens up possibilities for the two-parameter tuning of electronic properties by simple reversible mechanical deformations (Figure 45d). Additionally, this enables researchers to determine the dependence of the axial mechanical properties on torsional deformations. According to the results of [105], variations in Young’s modulus can reach 20–30 GPa in the *Q* region from 5 to 7 (Figure 45c).

**Figure 46:**
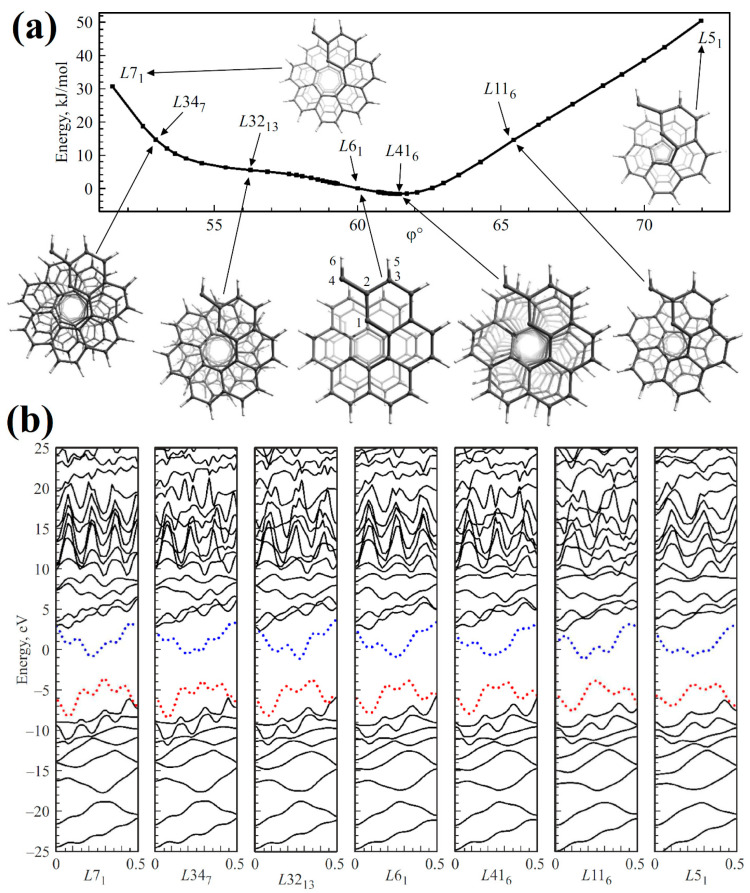
[$1_{zz}^{h}.1_{zz}^{h}$]helicene: (**a**) the energy diagram, top views of the atomic structures, and (**b**) electronic band structures in the helical Brillouin zone at different rotation angles, φ. The upper valence band is shown by a red dashed line; the lower conduction band is shown by a blue dashed line. Adapted from Ref. [108].

The electronic bands of [$1_{zz}^{h}.1_{zz}^{h}$]helicene in the helical Brillouin zone, obtained on the basis of DFT PBE0 calculations [108], change little as *Q* varies from 5 to 7 (Figure 46). This is due to the spring topology of nanohelicene, which retains its structure not only in the case of axial deformations but also in the case of torsion ones. In the latter case, the nanohelicene coils slide relative to each other, but the internal structure of the π-system is preserved.

**Figure 47:**
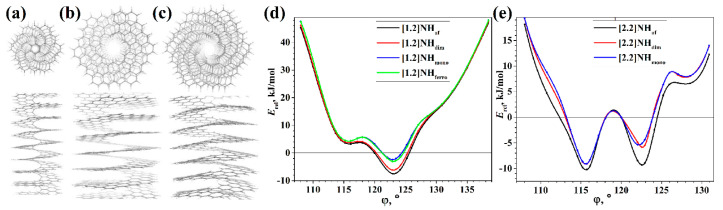
Top and side views of the global minima of (**a**) [$1_{zz}^{h}.2_{zz}^{h}$]helicene and (**b**) [$2_{zz}^{h}.2_{zz}^{h}$]helicene, and (**c**) the local minimum of about φ = 122.6° of [$2_{zz}^{h}.2_{zz}^{h}$]helicene. The energy diagrams of (**d**) [$1_{zz}^{h}.2_{zz}^{h}$]helicene and (**e**) [$2_{zz}^{h}.2_{zz}^{h}$]helicene. Reprinted from Ref. [112].

Further studies in the paradigm of helical periodicity were performed for [$m_{zz}^{h}.2_{zz}^{h}$]helicenes with *m* = 1 and 2 [112] (see Figure 47a–c). According to the results of [12], the first one (*m* = 1) is an anti-ferromagnetic semi-conductor. The second one (*m* = 2) is a diamagnetic metal that undergoes spontaneous symmetry-breaking due to Mott-Hubbard MIT under axial strain.

These results of [112] show that for such systems, it is necessary to carry out quantum chemical calculations of torsion energy curves for several states:

(i) highly symmetrical diamagnetic metallic;
(ii) diamagnetic semiconductor (Peierls MIT) with halved symmetry;
(iii) antiferromagnetic semiconductor (Mott-Hubbard MIT) with halved symmetry;
(iv) highly symmetrical ferromagnetic metallic.

The relationships between these four curves determine which MIT will be dominant in each concrete nanohelicene and in specific *Q* regions.

The torsion energy curves of [$1_{zz}^{h}.2_{zz}^{h}$]helicene show the preference for state (iii) over the entire torsion angle interval, and in the region of a single minimum, the difference between the energies of the states reaches maximal values (Figure 47d). When moving away from the minimum, the states become close in energy, which shows the principal possibility of the co-existence of these states under torsion.

The torsion energy curves of [$2_{zz}^{h}.2_{zz}^{h}$]helicene have a more complex structure, showing the presence of two minima that are very close in energy (Figure 47e). These minima are located on opposite sides of *Q* = 6 (or *Q’* = 3 for the corresponding states with halved symmetry), and state (iii) is most beneficial in them, which is the expected result.

We also note that in the intermediate region between the [$2_{zz}^{h}.2_{zz}^{h}$]helicene minima, the calculations show that all states are degenerate and belong to a state of type (i). The transition from one antiferromagnetic minimum to another leads to a reversible transformation through a diamagnetic metallic state. This provides the ability to construct promising electro-magneto-mechanical switches based on [$2_{zz}^{h}.2_{zz}^{h}$]helicene.

**Figure 48:**
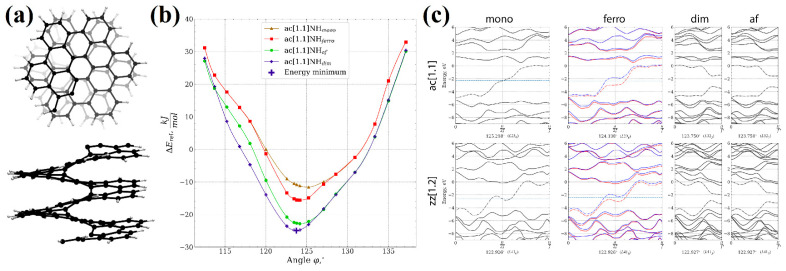
(**a**) Top and side views of the atomic structure of [$1_{ac}^{h}.2_{ac}^{h}$]helicene; (**b**) energy diagram of [$1_{ac}^{h}.2_{ac}^{h}$]helicene; (**c**) helical electronic bands of [$1_{ac}^{h}.2_{ac}^{h}$]helicene (marked as *ac*[1.1]) and [$1_{zz}^{h}.2_{zz}^{h}$]helicene (marked as *zz*[1.2]). Reprinted from Ref. [113] (Copyright 2023, with permission from Elsevier).

As was already mentioned [39], [$1_{ac}^{h}.2_{ac}^{h}$]helicene is metallic, which means that spontaneous symmetry-breaking can be expected from it. However, the preferred MIT type for this nanohelicene is not known a priori, which requires the calculation of four mentioned states, similar to [107]. The initially metallic [$1_{ac}^{h}.2_{ac}^{h}$]helicene and [$1_{zz}^{h}.2_{zz}^{h}$]helicene were studied in [113] in order to understand the effect of edge termination on the MIT type (Figure 48a). According to the results of [113], the lowest energy state for [$1_{ac}^{h}.2_{ac}^{h}$]helicene is state (ii), corresponding to Peierls MIT (see Figure 48b). Thus, the preference for one or another MIT is determined by the edge termination of nanohelicene.

The electronic bands in the helical Brillouin zone for nanohelicenes of both terminations were obtained for all states (Figure 48c). A half-populated helical band is intersected by the Fermi level, which leads to the metallic properties of state (i). Spontaneous symmetry-breaking leads to a gap in this helical band due to the folding of the helical Brillouin zone, which corresponds to dimerized states (ii) and (iii).

The study of the magnetic properties of nanohelicenes requires the consideration of spin effects in terms of magnetic line symmetry groups [102]. It is necessary to determine the partition of 81 families of magnetic line groups according to SST prototypes; this was carried out in [114]. We note that, according to the results of this work, the possibility of spin-splitting in the absence of spin-orbit interactions for nanohelicenes is also preserved when their symmetry is considered with the line groups.

## 7. Conclusions and Future Prospects

In conclusion, the current state of computational research of a promising class of one-dimensional nanostructures—nanohelicenes—is considered. Chirality, due to the intrinsic helical topology and the continuous π-system of these objects, leads to the manifestation of unique optical properties, circularly polarized luminescence (CPL), chirality-induced spin selectivity (CISS), and many others. To date, these properties have been studied only for molecular helicenes, but it is not difficult to assume that in the case of nanohelicenes, these properties will manifest themselves to an even greater extent. The helical topology leads to these properties having a significant dependence on reversible mechanical deformations. This is an important factor that makes these compounds promising for technological applications. To date, helicenes that are five–six coils in size have been experimentally synthesized. This shows the fundamental possibility of synthesizing such systems; however, it is obvious that the development of methods for the synthesis of nanohelicenes is at the beginning.

The results discussed in this review demonstrate that the properties of nanohelicenes are largely determined by the peripheral modification of the edges of the nanohelicene ribbon. To date, modifications leading to metallic or semiconductor nanohelicenes are known. Nanohelicenes can also have different magnetic ordering. To date, modifications with diamagnetic, antiferromagnetic, or ferromagnetic ordering have been found.

In order to classify nanohelicenes, we proposed a nomenclature based on the structure of graphene nanoflakes, from which nanohelicene is obtained using the idea of a Riemann surface. This nomenclature makes it possible to define the prototype classes of nanohelicenes and consider all other nanohelicenes as modifications to these classes.

One of the important consequences of helical topology is the significant dependence of the optical, electronic, and magnetic properties of nanohelicenes on mechanical deformations, in particular, axial ones. Recent theoretical works performed in the helical periodicity paradigm demonstrate that the properties of nanohelicene also strongly depend on torsional strains, and this dependence is more complex than that of axial considerations. It was shown that nanohelicenes are examples of nanostructures with helical periodicity only, which means that their symmetry is described by line groups with irrational orders of helical axes.

This review is devoted only to nanohelicenes built from carbon hexagons. However, there are known 1D structures in which some of the hexagons are replaced by other carbon polygons, such as squares, pentagons, heptagons, or octagons. Various combinations of these polygons open up a wide field for the modification of nanohelicenes, which is practically unexplored. Even broader prospects open up when some of the carbon atoms are replaced by hetero-atoms, for example, nitrogen, oxygen, sulfur, etc. Such systems have also practically not been studied. Such modifications lead to structures that will also have the basic properties of nanohelicenes, i.e., helical topology and continuous π-systems. In other words, nanohelicenes are just one of the classes of helical structures with a continuous π-system, the study of which will help to determine the future directions of research into such systems.

## Data Availability

No new data were created or analyzed in this study.
